# Sc(III) Complexes of 1,4,7-Triazacyclononane-1,4,7-triacetic Acid and Its Monoamides

**DOI:** 10.1021/acs.inorgchem.5c04142

**Published:** 2025-11-12

**Authors:** Jan Kubinec, Filip Koucký, Adam Svítok, Jan Faltejsek, Jan Kotek, Vojtěch Kubíček, Petr Hermann

**Affiliations:** † Faculty of Science, Department of Inorganic Chemistry, Charles University, Hlavova 8, 128 40 Prague, Czech Republic; ‡ Institute of Organic Chemistry and Biochemistry, Czech Academy of Sciences, Flemingovo náměstí 542/2, 160 00 Prague, Czech Republic

## Abstract

Scandium­(III) complexes with H_3_nota and its *N*-ethyl (H_2_ **L** ^1^) and *N,N*-diethyl (H_2_ **L** ^2^) monoamides were studied in the solid state and solution. Potentiometric measurements showed high stabilities of the binary Sc^III^ complexes with the ligands (log *K*_[Sc(L)]_ = 19.50, 16.64, and 17.94 for H_3_nota, H_2_ **L** ^1^ and H_2_ **L**,^2^ respectively) and weak coordination of the second ligand molecule under the ligand excess. The chemical model was confirmed by ^45^Sc NMR. Multinuclear NMR spectroscopy was used to study the ternary Sc^III^-ligand-oxalate systems. The results showed the presence of species with 2:1 and 1:1 [Sc­(L)]-to-oxalate stoichiometry. Ternary complexes with the nota-monoamides form two isomers differing in the position of oxalate versus amide pendant arm. In the solid state, the [Sc­(L)] complexes form oligomers interconnected through ligand carboxylate groups. Crystallization from solutions containing H_2_O_2_ or oxalate anions yielded ternary complexes. In all binary and ternary complexes, the Sc^III^ ion is octacoordinated with the N_3_O_4_O_1_ coordination mode. All of the carboxylate groups of the ligands are coordinated in the O_4_-plane. The apical position is occupied by an oxygen atom of water, peroxide, or carboxylate anion. The apical Sc–O bonds are usually longer compared to those of the oxygen atoms forming the O_4_-plane.

## Graphical abstract

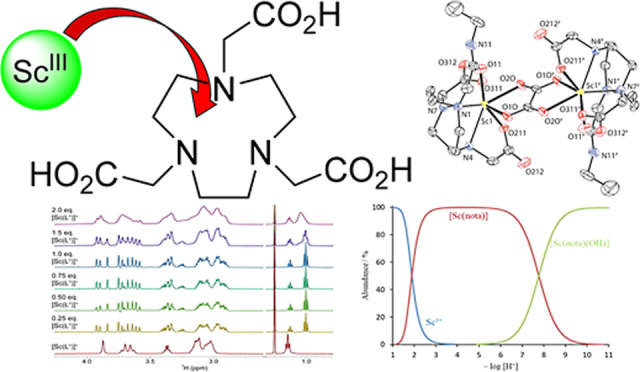

## Introduction

Radiodiagnosis and radiotherapy are important tools of modern medicine. Many radioisotopes are used in clinics, and even more are emerging ones. Significant attention has been recently devoted to radiopharmaceuticals based on scandium radioisotopes due to their suitable properties and increasing accessibility. Namely, ^43^Sc (*t*_1/2_ = 3.89 h) and ^44^Sc (*t*_1/2_ = 3.97 h) are the β^+^ emitters suitable for positron emission tomography, and ^47^Sc (*t*_1/2_ = 80.4 h) is the β^–^-emitter suitable for radiotherapy.

In medicine, metal ions cannot be used in a free form (i.e., as aqua ions) due to unspecific deposition in the tissues, typically sorption in bones or deposition in the liver. To prevent this, metal ions must be bound in thermodynamically stable and kinetically inert complexes which ensure their circulation in body fluids. Each metal ion requires, for stable coordination, specific ligands which match the metal ion size and chemical properties. Scandium exclusively forms a trivalent ion, and the ion is considered a hard Lewis acid. Thus, ligands used as Sc^III^ carriers mostly contain nitrogen and oxygen donor atoms. The Sc^III^ ion prefers coordination numbers from 6 to 8.

In recent years, most attention has been aimed at polyazamacrocyclic ligands as their complexes often show not only a high thermodynamic stability but also a high kinetic inertness, which further reduces the risk of radioisotope release and nonspecific deposition in the tissues.

Important chelator types used as metal radioisotope carriers are the derivatives of 1,4,7-triazacyclononane (tacn) and 1,4,7,10-tetraazacyclododecane (cyclen), mainly the acetate derivatives H_3_nota and H_4_dota (Figure 1), and their analogues bearing different pendant arms. The derivatives of both tacn and cyclen are also used as Sc^III^ carriers. Coordination properties of H_4_dota toward Sc^III^ ions have been investigated.^1^ Recently, the solid-state structure of Na­[Sc­(nota)­(CH_3_COO)] has been reported, and the ternary system Sc^III^–H_3_nota–CH_3_COO^–^ was studied experimentally by NMR and theoretically by DFT.^2^ However, a detailed solution study of the Sc^III^–H_3_nota system has not yet been reported yet. It is surprising as H_3_nota-analogues bearing picolinate pendant arm H_3_mpatcn and its phosphonate derivatives (Figure 1) have been recently suggested to be efficient carriers of scandium radioisotopes.^3–6^

**Figure 1:**
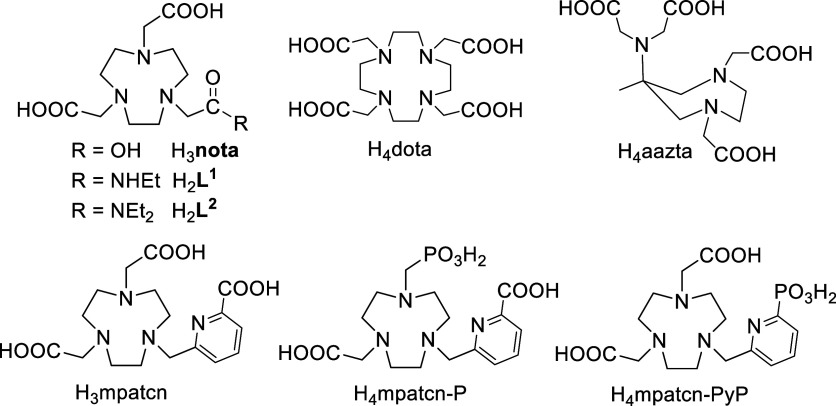
Discussed ligands.

Hydrophilic complexes with low-molecular weight, such as H_3_nota and H_4_dota complexes, show a nonspecific distribution in the tissues and a rapid renal clearance. Thus, attention is aimed at targeted radiotracers in which the complex is attached to a biomolecule (peptide, antibody, etc.), showing a high and selective affinity to the target tissue. The attachment of the targeting molecule requires the presence of a reactive group on the ligand molecule. The most common strategy is amide coupling using a pendant carboxylic acid group. Thus, nota- and dota-monoamides formed in the coupling reaction are used as metal radioisotope carriers. However, conversion of the carboxylic acid group participating in metal ion coordination into an amide changes the coordination properties of the ligand and the overall charge of the complex. Despite nota-monoamides having been used for many years as carriers of metal radioisotopes, the coordination properties of the ligands and their complexes have rarely been studied. The Ga^III^ complexes with methyl and benzyl monoamide form two isomeric complexes in which the amido group is coordinated by either oxygen or nitrogen atoms.^7^ Another study showed that the amide bound in the Ga^III^ complex with bis­(phosphonate)-bearing monoamide is hydrolyzed leading to cleavage of this bone-targeting group.^8^

Recently, our group published a detailed study reporting the chemical properties of H_3_nota and its coordination properties with Cu^II^ ion^9^ which was followed by a report on complexes of nota-monoamides with divalent metal ions.^10^ As a continuation of this work, here we report on a structural and solution study of Sc^III^ complexes with H_3_nota and its *N*-ethyl (H_2_ **L** **^1^**) and *N*,*N*-diethyl (H_2_ **L** **^2^**) monoamides (Figure 1). The ligands were chosen to investigate differences in the coordination behavior between the parent carboxylate ligand and its secondary and tertiary monoamides.

## Experimental Section

### Materials and Methods

Chemicals were purchased with synthetic purity from commercial sources and were used without purification. Ligands H_3_nota, H_2_ **L** **^1^**, and H_2_ **L** **^2^** were synthesized according to the published procedures.^9,10^ The ESI-MS and HPLC/MS measurements were performed on the Waters Arc HPLC system with UV/vis detector and Waters ACQUITY QDa mass detector with electrospray ionization and the quadrupole analyzer. The HPLC analyses were performed on the Cortecs C18 (2.7 μm, 4.6 × 50 mm) column using a flow of 1.2 mL/min of the 0.1% TFA in H_2_O and 0.1% TFA in acetonitrile (MeCN) mixture according to the gradient: 0.0 min 0% MeCN, 0.5 min 5% MeCN, 3.5 min 100% MeCN, 4.0 min 100% MeCN.

### Potentiometry

Potentiometry was carried out according to the previously published procedures; for the preparation of stock solutions and chemicals, equipment, electrode system calibration, titration procedures and data treatment, see refs 11–13 . Throughout the paper, pH means −log­[H^+^]. The protonation and stability constants were determined in 0.1 m (NMe_4_)Cl at 25.0 °C with p*K*_w_ = 13.81. The Sc^III^–H_3_nota system was studied by in-cell titrations from data obtained in the pH range 1.6–12 with ∼40 points per titration and four parallel titrations (*c*_L_ = 0.004 m, *c*_Sc_ = 0.004 or 0.002 m). The Sc^III^–H_2_ **L** **^1^** and Sc^III^–H_2_ **L** **^2^** systems were studied by a combination of the out-of-cell and in-cell methods. The out-of-cell method was performed at 1:1 ligand-to-metal ratio (*c*_L_ = *c*_Sc_ = 0.004 m, pH 1.5–5.0, 15–25 points per titration), and the in-cell method was performed at 1:1 and 2:1 ligand-to-metal ratios (*c*_L_ = *c*_Sc_ = 0.004, pH 5.0–12.0 or until precipitation of hydroxide, 15–20 points per titration; or *c*_L_ = 0.004 m, *c*_M_ = 0.002 m, pH 2.0–11.5, ∼40 points per titration). The stability constants were calculated with OPIUM program,^14^ and the presented chemical models were chosen to have a chemical sense and exhibit the best-fit statistics. Stability constants for the Sc^III^–OH^–^ system were taken from the literature.^15^ Protonation constants of ligands were adopted from literature—H_3_nota: 13.17, 5.74, 3.22, 1.96, 0.70;^9^ H_2_ **L** **^1^**: 13.03, 4.24, 2.04, 0.67;^10^ H_2_ **L** **^2^**: 12.93, 4.91, 2.48, 0.92.^10^

### Syntheses of Complexes

#### Binary Sc^III^-Complexes

Corresponding ligand (0.30 mmol) and ScCl_3_·6H_2_O (0.33 mmol) were dissolved in water (2 mL) and 0.1% aq. LiOH was added portion-wise to reach pH 6 during 3 h. The metal ion excess precipitated as hydroxide. The precipitate was filtered off with a 0.22 μm syringe filter, and the pH of the filtrate was adjusted to 4 with 0.1% aq. HCl. The volatiles were evaporated in vacuo, and the solid residue was redissolved in 0.5 M aqueous LiClO_4_ (∼2 mL). The crystalline complex was obtained by diffusion of *i*PrOH vapors into the solution. Crystals were collected by filtration, washed with EtOH, and dried in air. The NMR spectra of the complexes are shown in Figures S1–S3.

#### [Sc­(nota)]·2H_2_O

Yield 75%; NMR: ^1^H (600 MHz, H_2_O + D_2_O, pH 7.0): δ 3.68 (s, 6H, C**H**_**2**_CO_2_), 3.22–2.95 (m, 12H, macrocyclic ring C**H**_**2**_); ^13^C­{^1^H} (151 MHz, H_2_O + D_2_O, pH 7.0): δ 180.2 (CH_2_ **C**O_2_), 65.9 (**C**H_2_CO_2_), 55.4 (macrocyclic ring **C**H_2_); ^45^Sc (97 MHz, H_2_O + D_2_O, pH 7.0): δ 83.3 (*w*_1/2_ 1670 Hz); MS­(+): 345.91 [ML + H]^+^ (calcd 346.08); Elemental analysis: found (calcd for C_12_H_22_N_3_O_8_Sc): C, 37.52 (37.80) H, 5.91 (5.82) N, 11.04 (11.02).

#### [Sc­(**L^1^**)]­ClO_4_·4H_2_O

Yield 73%; NMR: ^1^H (600 MHz, H_2_O + D_2_O, pH 7.0): δ 3.87 (s, 2H, C**H**_**2**_CON), 3.68 (pseudo q (AB system), ^3^ *J*_HH_ 16.7, 4H, C**H**_**2**_CO_2_), 3.37 (q, ^3^ *J*_HH_ 8.0, 2H, NHC**H**_**2**_CH_3_), 3.25–2.86 (m, 12H, macrocyclic ring C**H**_**2**_), 1.15 (t, ^3^ *J*_HH_ 7.9, 3H, CH_2_C**H**_**3**_); ^13^C­{^1^H} (151 MHz, H_2_O + D_2_O, pH = 7.0): δ 180.2 (CH_2_ **C**O_2_), 175.1 (CH_2_ **C**ON), 65.8 (**C**H_2_CO_2_), 63.5 (**C**H_2_CON), 55.6, 55.3, and 55.1 (all macrocyclic ring **C**H_2_), 36.4 (NH**C**H_2_CH_3_), 13.8 (CH_2_ **C**H_3_); ^45^Sc (97 MHz, H_2_O + D_2_O, pH 7.0); δ 82.7 (*w*_1/2_ 1830 Hz); MS­(+): 373.31 [ML]^+^ (calcd 373.13); Elemental analysis: found (calcd for C_14_H_32_ClN_4_O_13_Sc): C, 30.95 (30.86) H, 5.79 (5.92) N, 10.32 (10.28).

#### [Sc­(**L^2^**)]­ClO_4_·5H_2_O

Yield 70%; NMR: ^1^H (600 MHz, H_2_O + D_2_O, pH 7.0): δ 4.10 (s, 2H, C**H**_**2**_CON), 3.67 (pseudo dd (AB system), *J*_HH_ 39.9, 14.9, 4H, C**H**_**2**_CO_2_), 3.56–3.33 (m, 4H NC**H**_**2**_CH_3_), 3.25–2.94 (m, 12H, macrocyclic ring C**H**_**2**_), 1.21 (t, ^3^ *J*_HH_ 7.7, 3H, CH_2_C**H**_**3**_), 1.19–1.11 (m, 3H, NCH_2_C**H**_**3**_); ^13^C­{^1^H} NMR (151 MHz, H_2_O + D_2_O, pH 7.0): δ 180.2 (CH_2_ **C**O_2_), 173.9 (CH_2_ **C**ON), 65.8 (**C**H_2_CO_2_), 62.7 (**C**H_2_CON), 55.9–55.0 (macrocyclic ring **C**H_2_), 44.0 and 43.2 (both N**C**H_2_CH_3_), 13.6 and 12.7 (both CH_2_ **C**H_3_); ^45^Sc NMR (97 MHz, H_2_O + D_2_O, pH 7.0): δ 81.6 (*w*_1/2_ 2320 Hz); MS­(+): 401.35 [ML]^+^ (calcd 401.16); Elemental analysis: found (calcd for C_16_H_38_ClN_4_O_14_Sc): C, 32.40 (32.52); H, 6.51 (6.48); N, 9.35 (9.48).

### Ternary Sc^III^ Complexes

Oxalic acid dihydrate (0.30 mmol) or 30% aq. H_2_O_2_ (1.5 mmol) was added to the binary complex (0.30 mmol) in an aqueous solution (15 mL). pH was adjusted to 6 with aq. LiOH (∼0.1%), and the solution was stirred overnight. Then, the solution was layered with MeOH (∼3 mL) and colorless crystals were obtained by a slow diffusion of acetone vapors into the solution. The single crystals used for the X-ray diffraction analysis were selected from the bulk material, and the compositions obtained from elemental analysis correspond well to those obtained from the crystal structures. However, the complexes form a mixture of species in solution; thus, their NMR signals are not listed here (see section Results and Discussion). Some selected NMR spectra of freshly prepared solutions of the ternary complexes with oxalate anions are shown in Figures S4–S6.

#### Li_2_[{Sc­(nota)}_2_(O_2_)]·10H_2_O

Yield: 30%; EA: found (calcd for C_24_H_56_Li_2_N_3_O_24_Sc_2_): C, 31.42 (31.45) H, 6.32 (6.16) N, 9.02 (9.17).

#### [{Sc­(**L^1^**)}_2_(O_2_)]·10H_2_O

Yield: 43%; EA: found (calcd for C_28_H_68_N_8_O_22_Sc_2_): C, 34.98 (35.08) H, 7.10 (7.15) N, 11.80 (11.69).

#### [{Sc­(**L^2^**)}_2_(O_2_)]·12H_2_O

Yield: 62%; EA: found (calcd for C_32_H_80_N_8_O_24_Sc_2_): C, 36.55 (36.57) H, 7.80 (7.67) N, 10.62 (10.66).

#### Li_6_[{Sc­(nota)}_3_(C_2_O_4_)_3_]·14H_2_O

Yield: 25%; EA: found (calcd for C_42_H_82_Li_6_N_9_O_44_Sc_3_): C 31.40 (31.65), H 5.27 (5.19), N 7.99 (7.91).

#### [{Sc­(**L^1^**)}_2_(C_2_O_4_)]·10H_2_O

Yield: 75%; EA: found (calcd for C_30_H_68_N_8_O_24_Sc_2_): C, 35.56 (35.51) H, 6.84 (6.75) N, 10.89 (11.04).

### NMR Spectroscopy

The NMR experiments were carried out on the Bruker Avance III 600 (with a cryoprobe), Bruker Avance III HD 400 or Varian VNMRS 300 MHz or Varian Unity Inova-400 spectrometers in 5 mm tubes. Chemical shifts are given in ppm, and the coupling constants are in Hz. All NMR spectra were collected at 25 °C. The shifts were referenced with external standards: 0.1% *t*BuOH in D_2_O (^1^H δ 1.25 ppm, ^13^C δ 30.30 ppm). External reference for ^45^Sc was 0.1 M solution of Sc­(ClO_4_)_3_ in 1 M aq. HClO_4_.^1^ The pH of samples was adjusted with diluted aq. HCl or aq. LiOH (∼0.5%), or maintained with 0.25 M imidazole/HCl buffer. Binary complexes were prepared by mixing solid ligands (typically at a final concentration of 25 mM) with standardized ScCl_3_ solution in Sc/L 1:1 or 1:2 molar ratios. Ternary complexes with oxalate anions were studied by mixing a lithium­(I) oxalate aq. solution (25 mM) and variable amounts of stock solutions (200 mM) of Sc^III^ complexes with the studied ligands.

### X-ray Diffraction

The single crystals of the binary complexes [Sc­{Sc­(nota)}_6_]­Cl_3_·26H_2_O and Li_3_[{Sc­(**L** ^1^)}_6_]_2_(ClO_4_)_10_Cl_5_·6Me_2_CO·35.5H_2_O used for X-ray diffraction analysis were prepared as follows: the bulk complex (10 mg) was dissolved in H_2_O or 0.25 m aq. LiClO_4_ (∼2 mL), and the pH of the solutions was adjusted to 2.5 or 5, respectively, with aq. HCl or aq. LiOH (∼0.1%). The complexes crystallized upon diffusion of acetone vapors into the solution. The single crystals of the ternary complexes Li_2_[{Sc­(nota)}_2_(O_2_)]·10H_2_O, [{Sc­(**L** ^1^)}_2_(O_2_)]·10H_2_O, [{Sc­(**L** ^2^)}_2_(O_2_)]·12H_2_O, Li_6_[{Sc­(nota)}_3_(C_2_O_4_)_3_]·14H_2_O and [{Sc­(**L** ^1^)}_2_(C_2_O_4_)]·10H_2_O used for X-ray diffraction analysis were selected from the bulk material isolated as described above.

The selected crystals were mounted on a glass fiber in a random orientation, and diffraction data were collected using a Bruker D8 VENTURE Duo diffractometer at 120 K with a microfocus sealed tube employing Mo Kα radiation (λ 0.71073 Å). Data were analyzed with the SAINT software package (SAINT V8.40B, Bruker AXS Inc., 2019). Absorption effects were corrected using the multiscan method (SADABS).^16^ All structures were solved by direct methods (SHELXT2018)^17^ and refined using full-matrix least-squares techniques (SHELXL2017).^18^ Generally, all non-hydrogen atoms were refined anisotropically. Hydrogen atoms were located in the electron density map; however, those attached to carbon atoms were positioned theoretically using *U*_eq_(H) = 1.2 *U*_eq_(C) to decrease the number of refined parameters, while hydrogen atoms bonded to heteroatoms (N and O) were fully refined. Some hydrogen atoms belonging to O–H or N–H groups were fixed in their original or theoretical positions if full refinement resulted in unrealistically short or long bond distances.

In the crystal structure of [Sc­{Sc­(nota)}_6_]­Cl_3_·26H_2_O, the independent part corresponds to one-sixth of the formula unit due to trigonal space group *R*3̅ symmetry. The independent Sc­(nota) unit was refined considering a disorder of two macrocycle CH_2_ groups and one pendant CH_2_ group into two tied positions with occupancies of 61% and 39%. Many weak and closely spaced electron maxima in the difference density map indicated numerous disordered water molecules, which were treated using the SQUEEZE command by Platon;^19^ the squeezed density corresponds to 23 water molecules per formula unit. Some remaining maxima were interpreted as chloride ions (one with a crystallographic occupancy of 1/3 and another with 1/6) and the half-occupied water molecule.

In the crystal structure of Li_3_[{Sc­(**L** ^1^)}_6_]_2_(ClO_4_)_10_Cl_5_·6Me_2_CO·35.5H_2_O, the independent part corresponds to one-half of the formula unit. Due to a centrosymmetry of the space group, there are two-halves of two [{Sc­(**L** ^1^)}_6_]^6+^ hexameric units in the structurally independent part, *i.e*., six independent {Sc­(**L** ^1^)}^+^ complexes in total. Several CH_2_ groups and macrocycle nitrogen atoms of three {Sc­(**L** ^1^)}^+^ complexes were treated as disordered into two positions, as well as two ethylamido groups of the pendant arms. In the centers of the [{Sc­(**L** ^1^)}_6_]^6+^ hexamer cavities, the [Li­(H_2_O)_4_]^+^ complexes are present, one in full and the other with half-occupancy. As the counterions, five structurally independent perchlorate ions were found, and two of them were refined with a disorder of the oxygen atoms sharing a central chlorine atom. In addition, three chloride ions were localized, one of them with half occupancy. Three molecules of acetone were localized, as well as five other intense maxima attributable to water molecules of crystallization. However, the corresponding hydrogen atoms could not be found. Other remaining number of weak and closely spaced electron maxima indicated numerous disordered water molecules, which were refined with low occupancies to obtain a reliable total occupancy in given subspaces.

In the crystal structures of Li_2_[{Sc­(nota)}_2_(O_2_)]·10H_2_O, [{Sc­(**L** ^1^)}_2_(O_2_)]·10H_2_O, and [{Sc­(**L** ^2^)}_2_(O_2_)]·12H_2_O, the independent parts correspond to one-half of the formula units. The basic structural motive is the same in all three crystal structures; a dimer is formed through the center of symmetry lying at the midpoint of the oxygen–oxygen bond of the peroxide ligand. No disorder was found in the case of Li_2_[{Sc­(nota)}_2_(O_2_)]·10H_2_O. However, in the structure of [{Sc­(**L** ^1^)}_2_(O_2_)]·10H_2_O, the terminal methyl group of the amidoethyl moiety was split into two positions in the final refinement, and oxygen atoms of two water molecules of crystallization lay in the special positions with half-occupancy. In the structure of [{Sc­(**L** ^2^)}_2_(O_2_)]·12H_2_O, the oxygen atoms of two water molecules were refined as disordered into two closely spaced positions sharing corresponding hydrogen atoms.

In the crystal structure of Li_6_[{Sc­(nota)}_3_(C_2_O_4_)_3_]·14H_2_O, the independent part corresponds to the declared formula unit. One carboxylic group of oxalate was refined as a split into two positions. Eleven water molecules of crystallization were located in the electron density map (some of them coordinated to the Li^I^ ions), and one of them was refined as disordered in two positions, sharing one hydrogen atom. Some remaining weak and closely spaced electron maxima, indicating a few disordered water molecules, were squeezed using Platon;^19^ the corresponding density was equal to other 3 water molecules per formula unit.

In the crystal structure of [{Sc­(**L** ^1^)}_2_(C_2_O_4_)]·10H_2_O, the independent part corresponds to one-half of the formula unit, and the complex forms a centrosymmetric dimer through the midpoint of the carbon–carbon bond of the oxalato ligand. A notable disorder was observed in the macrocyclic part. The disorder resembles those scarcely found in the complexes of dota-like ligands.^20–22^ It was addressed by splitting all atoms of the macrocycle into two positions with relative occupancies of 73% and 27%, respectively. Pairs of split positions of the nitrogen atoms bearing the acetate pendant arms are very close to each other, and the pendant arms were treated with no disorder. However, the *N*-ethyl-acetamido pendant was split into two positions, sharing only the coordinated oxygen atom. The disorder is shown in Figure S7. From several water molecules of crystallization, the oxygen atoms of two of them lie in the special positions with half-occupancy.

Crystallographic parameters are given in Table S3.

### DFT Calculations for [Sc­(L^1,2^)­(ox)]^−^ Complex Isomers

The calculations employed the GGA functional BP86,^23,24^ Grimme’s D3­(Becke-Johnson) dispersion correction,^25,26^ and a def2-TZVP^27^ basis set for main group elements and def2-TZVP basis set for Sc^III^ as implemented in the TURBOMOLE v7.6 code.^28^ The structures of all considered metal–ligand variations with fully saturated coordination spheres were optimized at ambient temperature in the COSMO implicit solvent model.^29,30^ The global energy minima of the oxalate ligand coordination were investigated depending on the geometry of the pendant arms. All calculations were performed by using the PCM model (ε = 80) in water. Due to the fully saturated coordination sphere of all studied complexes, no calculations with explicitly defined water molecules were conducted. The initial geometries for the calculations were taken from the X-ray structures.

## Results and Discussion

### Syntheses and Solid-State Structures of the Binary Complexes

The Sc^III^ complexes with H_3_nota, H_2_ **L** ^1^, and H_2_ **L** ^2^ were prepared by reacting an excess of ScCl_3_·6H_2_O with the ligand upon slow addition of aq. LiOH until the solution’s pH remained steady at 6. The excess Sc^III^ precipitated as a hydroxide and was separated by filtration. Characterization NMR spectra of the complexes are shown in Figures S1–S3.

The single crystals suitable for X-ray analysis were grown by vapor diffusion of an organic solvent (MeOH, *i*PrOH, acetone) into the aqueous solutions of the complexes. The crystallizations were carried out at various pH values and in the presence of anions compensating for the positive charge of the complexes.

Crystallization of [Sc­(nota)] from aqueous solution at pH ∼ 2.5 yielded single crystals of [Sc­{Sc­(nota)}_6_]­Cl_3_·26H_2_O; the extra Sc^III^ ion was released from the complex due to standing at low pH for a long time. Thus, such a crystalline phase is a product of partial decomplexation. Due to a crystallographic symmetry, only one {Sc­(nota)} unit is structurally independent. The Sc^III^ ion of the {Sc­(nota)} unit is octacoordinated in the N_3_O_4_O_1_ coordination environment (Figure 2, Table S1). The ligand coordinates by three amine nitrogen atoms (*d*_Sc–N_ = 2.36–2.39 Å) in the basal N_3_ plane and three carboxylate oxygen atoms (*d*_Sc–O_ = 2.10–2.22 Å) in the equatorial O_4_ plane. The last position of the equatorial plane (*d*_Sc–O_ = 2.21 Å) and the apical position (*d*_Sc–O_ = 2.44 Å) are occupied by two oxygen atoms of one carboxylate group of the neighboring {Sc­(nota)} unit. Three {Sc­(nota)} units related by 3-fold symmetry are connected in such a way to form a cyclotrimer that facially coordinates the extra Sc^III^ ion, which occupies a position with a crystallographic occupancy of 1/6, through the carboxylate oxygen atoms (*d*_Sc–O_ = 2.09 Å). The other symmetry-related cyclotrimer coordinates the opposite side of the extra Sc^III^ ion, creating an octahedral environment. So, the extra Sc^III^ ion serves as a template for the formation of the hexameric {Sc­(nota)}_6_ superstructure (Figure 2).

**Figure 2:**
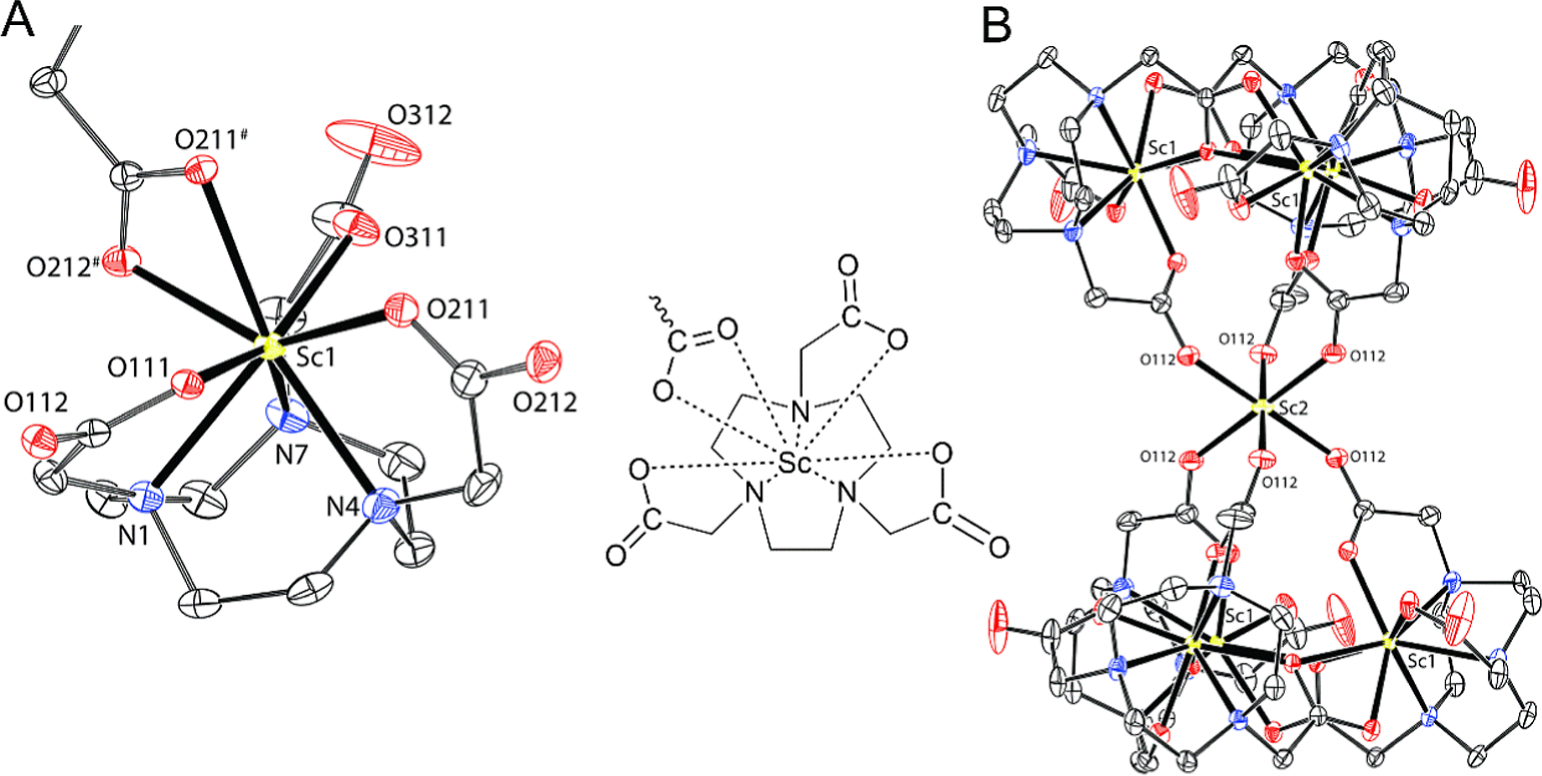
Crystal structure of [Sc­{Sc­(nota)}_6_]­Cl_3_·26H_2_O. The molecular structure of the {Sc­(nota)} unit (A; the κ-O,O*′-*coordinated carboxylate with atoms O211# and O212# belongs to the neighboring {Sc­(nota)} unit). The [Sc­{Sc­(nota)}_6_]^3+^ cation (B). The hydrogen atoms and charges are not shown for clarity.

Crystallization of the [Sc­(**L** ^1^)]^+^ complex from aqueous solution containing LiClO_4_ at pH 5 by a slow diffusion of acetone yielded single crystals of composition Li_3_[{Sc­(**L** ^1^)}_6_]_2_(ClO_4_)_10_Cl_5_·6Me_2_CO·35.5H_2_O. Similar to the previous structure, the Sc^III^ ions of all {Sc­(**L** ^1^)} units are octacoordinated in the N_3_O_4_O_1_ coordination environment, here formed by the basal N_3_ plane of three macrocycle nitrogen atoms, the equatorial O_4_ plane formed by three oxygen atoms of the pendant arms and one oxygen atom of the carboxylate group from the neighboring complex, and one water molecule in the apical position (Figure 3). The interconnection of the units creates a centrosymmetric cyclohexameric structure with three structurally independent {Sc­(**L** ^1^)} units (Figure 3). In the crystal structure, there are two such hexamers. Their structures are further stabilized by a system of hydrogen bonds to tetrahedral [Li­(H_2_O)_4_]^+^ complex cations placed in the hexamer’s cavity. However, geometries of all {Sc­(**L** ^1^)} units are analogous and, thus, only one representative complex is shown in Figure 3. The geometries are similar to those found in the Sc^III^–H_3_nota complex described above, with *d*_Sc–N_ = 2.34–2.48 Å, *d*_Sc–O_ = 2.11–2.25 Å for the O_4_ planes, and *d*_Sc–O_ = 2.26–2.31 Å for the apical water molecules (Table S1).

**Figure 3:**
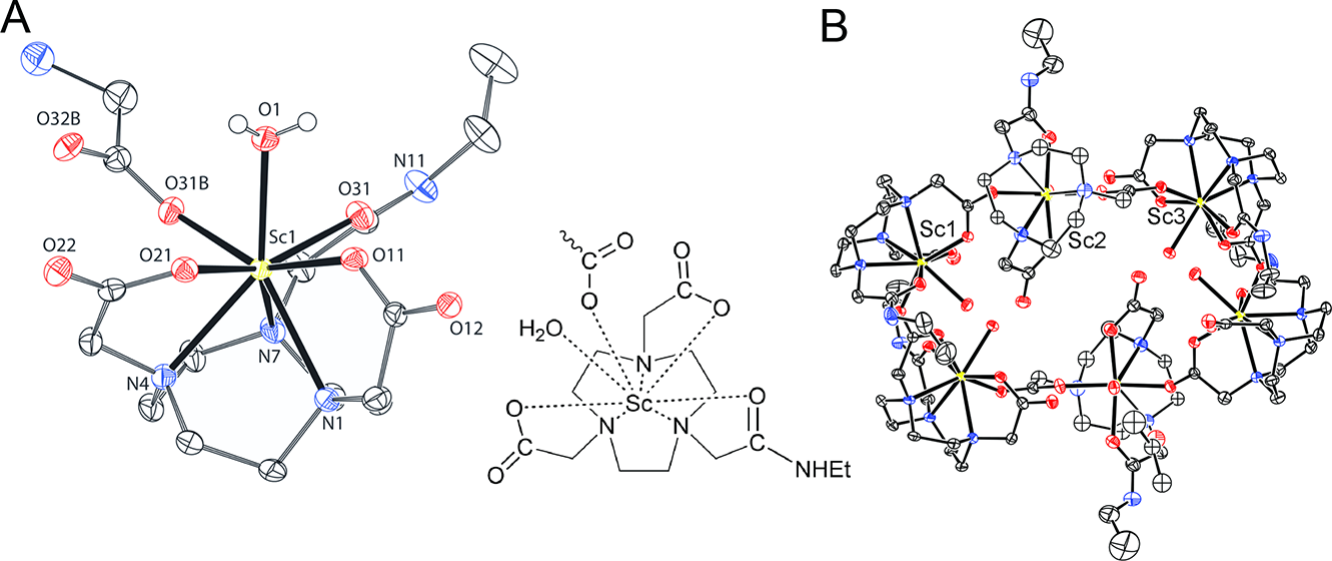
Crystal structure of Li_3_[{Sc­(**L** ^1^)}_6_]_2_(ClO_4_)_10_Cl_5_·6Me_2_CO·35.5H_2_O. The molecular structure of a representative {Sc­(**L** ^1^)­(H_2_O)}^+^ unit (A; the additional carboxylate coordinated through oxygen atom O31B comes from the neighboring {Sc­(**L** ^1^)} unit). One of the hexameric complex cations [{Sc­(**L** ^1^)}_6_]^6+^ is presented in the crystal structure (B). The most of hydrogen atoms and charges are not shown for clarity.

### Thermodynamic Stability

High thermodynamic stability of complexes is an important property of metal complexes used in radio medicine. Thus, stability constants of the Sc^III^ complexes with the studied chelators were determined by potentiometry. Complexation of Sc^III^ by H_3_nota was fast, and thus, it was studied by the direct titration technique (in-cell method). Complexation of Sc^III^ by H_2_ **L** ^1^ and H_2_ **L** ^2^ was slower in the acidic region (pH < 3) and, thus, the batch titrations (out-of-cell method) had to be applied. In addition, the systems were studied in-cell in the neutral and weakly alkaline regions. The strongly alkaline region cannot be studied in the Sc^III^–H_2_ **L** ^1^ and Sc^III^–H_2_ **L** ^2^ systems due to decomposition of the complexes and precipitation of Sc^III^ hydroxide. Examples of the titration curves are shown in Figures S8 and S9.

The [Sc­(L)] species (through the following text, general “L” represents all studied ligands, and the charge of the species will not be specified) are dominant in the acidic and neutral regions in all systems (Figure 4). The Sc^III^ ion is mostly octacoordinated in complexes with polydentate ligands. Thus, H_3_nota and its monoamides do not saturate the coordination sphere and two water molecules are probably coordinated to the central metal ion in solution.^31^ The water molecules can be easily deprotonated, and thus, the monohydroxido species are formed in the neutral region (log *K*_a_ = 7.4–7.8). In addition, [Sc­(HL)­(L)] species were found in all systems with the ligand excess. However, coordination of the second ligand molecule is rather weak (log *K* = 2.9–3.2, Table 1) as the second ligand molecule is probably coordinated only through one or two carboxylate groups, similar to the coordination motifs found in the solid state (see above).

**Figure 4:**
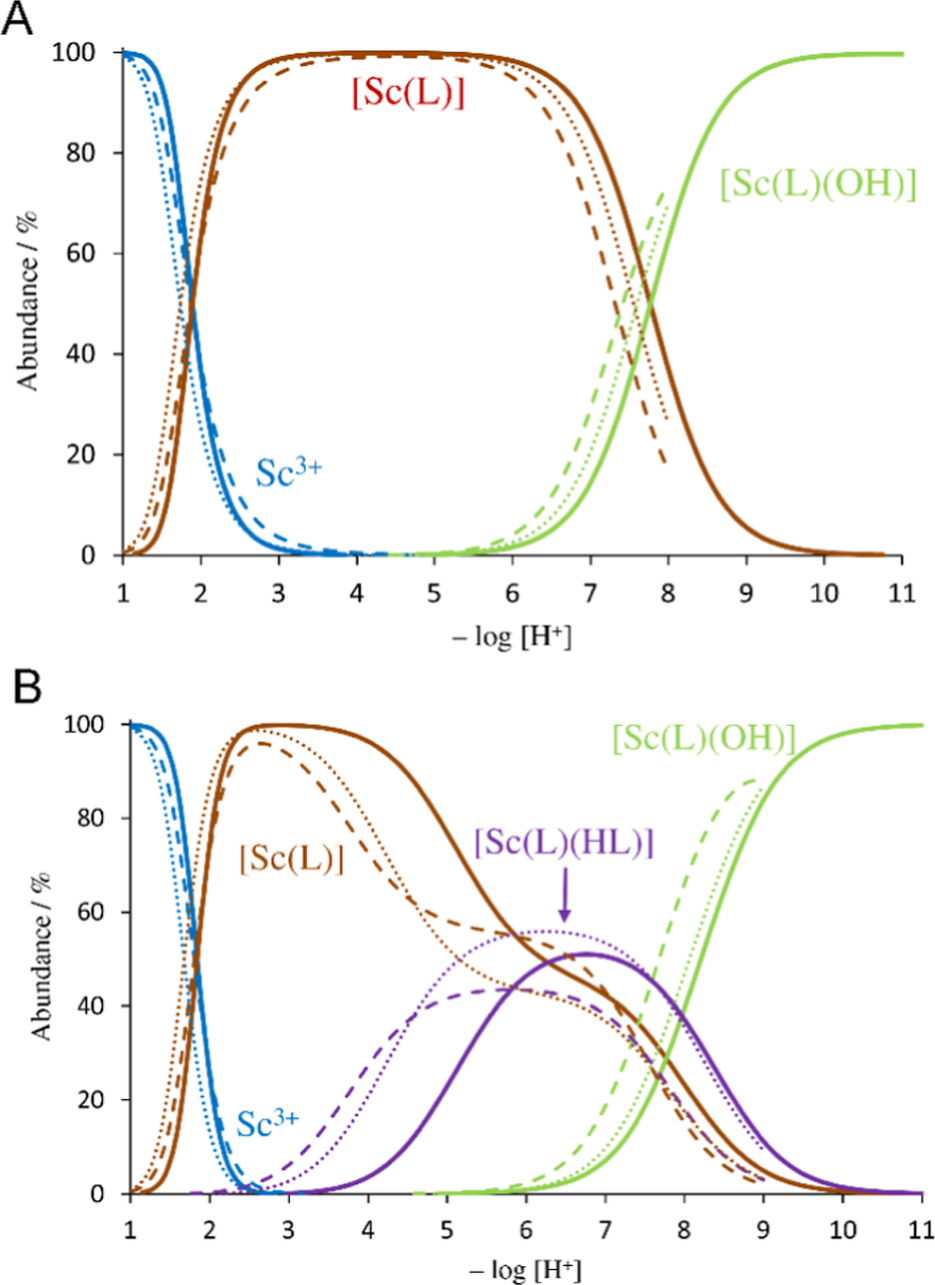
Distribution diagrams of the Sc^III^–H_3_nota (solid line), Sc^III^–H_2_ **L^1^** (dashed line), and Sc^III^–H_2_ **L^2^** (dotted line) systems ((A) *c*_L_ = *c*_M_ = 4 mM, (B) *c*_L_ = 4 mM, *c*_M_ = 2 mM; *I* = 0.1 M (NMe_4_)­Cl, 25 °C).

**Table 1:** Equilibrium Constants (log *K*) of Sc^III^ Complex with the Studied Ligands (*I* = 0.1 m NMe_4_Cl, 25 °C)^a^ ^,^ ^b^

equilibrium	H_3_nota	H_2_ **L** ^1^	H_2_ **L** ^2^
Sc + L ↔ [Sc(L)]	19.50	16.64	17.94
[Sc(L)(OH)] + H+ ↔ [M(L)(H2O)]	7.77	7.36	7.58
[Sc(L)] + HL ↔ [M(HL)(L)]	3.11	2.86	3.19

a Charges are omitted. Ligand p*K*_a_’s: H_3_nota: 13.17, 5.74, 3.22, 1.96, 0.70;^9^ H_2_ **L** ^1^: 13.03, 4.24, 2.04, 0.67;^10^ H_2_ **L** ^2^: 12.93, 4.91, 2.48, 0.92.^10^

b Overall stability constants are listed in Table S4.

The [Sc­(L)] species show high thermodynamic stabilities (log *K* = 16.6–19.5). The determined stability constants of the [Sc­(**L** ^1^)]^+^ and [Sc­(**L** ^2^)]^+^ complexes are lower than the stability constant of the [Sc­(nota)] complex due to the decreased coordination ability of the amide carbonyl group. As Sc^III^ ion is too large for the H_3_nota cavity, the values of stability constants are lower than those determined for Ga^III^, Cu^II^, and Zn^II^ ions and are comparable to stabilities of complexes of large metal ions such as Cd^II^ or Pb^II^ (Table 2).^9^ The low number of donor atoms in all studied ligands also results in less stable Sc^III^ complexes than those of octadentate ligands [Sc­(dota)]^−^ (log *K*_[M(L)]_ = 30.79),^1^ [Sc­(dtpa)]^2–^ (log *K*_[M(L)]_ = 27.43),^1^ [Sc­(aazta)]^−^ (log *K*_[M(L)]_ = 27.69),^32^ and [Sc­(mpatcn)] and its phosphonate analogues H_4_mpatcn-P and H_4_mpatcn-PyP (log *K*_[M(L)]_ = 21–26);^5^ for structural formulas of all ligands, see Figure 1.

**Table 2:** Comparison of Stability Constants log *K*_ML_ of Studied Complexes with Sc^III^ and Other Metal Ions.^9,10,33^

metal ion	H_3_nota	H_2_L^1^	H_2_L^2^
Sc^III^	19.50	16.63	18.73
Ga^III^	29.60		
Cu^II^	23.33	21.37	22.72
Zn^II^	22.32	18.89	19.30
Cd^II^	17.87		
Pb^II^	18.18		

### Solution NMR Study

To confirm speciation determined by potentiometry and to get better insight into the solution behavior of the complexes, the binary systems Sc^III^–H_3_nota and its monoamides were studied in detail by ^45^Sc and ^1^H NMR (Figures 5 and S10–S13).

**Figure 5:**
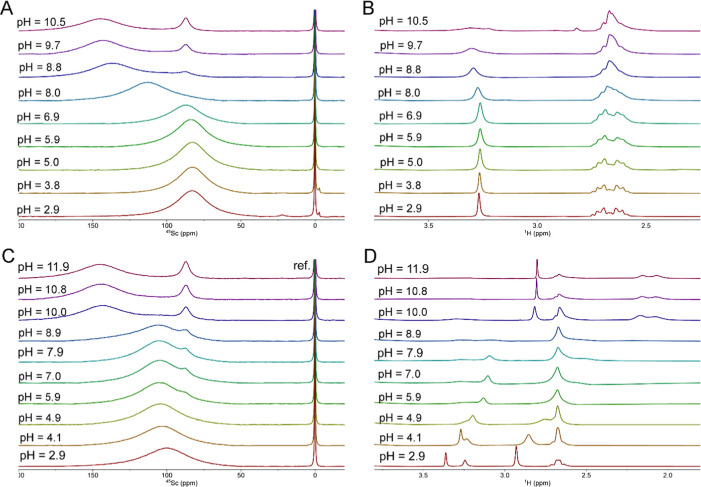
^45^Sc (A,C) and ^1^H (B, D) NMR spectra of the Sc^III^–H_3_nota system at 1:1 (A,B) and 1:2 (C,D) Sc^III^-to-H_3_nota ratios (*c*_Sc_ = 25 mM, 25 °C).

In acidic solutions with 1:1 stoichiometry, a broad intensive ^45^Sc NMR signal of the [Sc­(L)] complexes at 75–85 ppm is present together with a small narrow signal of free Sc^III^ ion centered at 7 ppm which fully disappears at pH > 3, consistently with the distribution diagram. Based on the solid-state structures, the complexes in aqueous solution likely coordinate two water molecules. In the neutral region, the ^45^Sc NMR signal of the complex shifts to 140–150 ppm due to deprotonation of the coordinated water molecule and formation of the monohydroxido complexes. In parallel, another signal at 90 ppm appears in the alkaline region. This signal is significantly narrower than the former species, which indicates a more symmetrical or rigid structure. These two signals remain in the alkaline solution, where—according to potentiometry—only the monohydroxido complex should be present. The presence of these two signals can be explained by an equilibrium between monomeric [Sc­(L)­(OH)] and dimeric [Sc_2_(L)_2_(μ–OH)_2_] forms; the double-hydroxide-bridged dimers are a common motif in the Sc^III^ coordination chemistry.^34–37^ However, such speciation cannot be evaluated from the potentiometric data as there is no change in the overall protonation state per one Sc^III^ ion. The ^1^H NMR spectra of [Sc­(nota)] show well-resolved sets of macrocyclic CH_2_ signals in the acidic solution, pointing to a decreased symmetry, in comparison with the parent ligand due to the coordination of the metal ion. The signals of pendant CH_2_ groups are broad singlets, pointing to fast pendant arm rotations. In the case of [Sc­(**L** ^1^ **/L** ^**2**^)]^+^ complexes, the signals of pendant CH_2_ groups are split to AB-doublets. Formation of the hydroxido species in the alkaline region leads to significant ^1^H NMR signal broadening, which could be ascribed to the dynamic equilibrium between the monomeric and dimeric species.

All species discussed above were also identified in the ^45^Sc NMR spectra at the 1:2 Sc^III^-to-ligand ratio in alkaline solutions (unfortunately, the system Sc^III^–H_2_ **L** ^1^ cannot be efficiently studied above pH 8 due to precipitate formation). In acidic solutions, the signal of the free Sc^III^ ion is very small or even not present, and the broad signals of the complexes are centered at somewhat higher chemical shift (100–110 ppm) compared to the 1:1 solutions. It can be attributed to the formation of the 2:1 complex [Sc­(L)­(HL)], in which one ligand is fully coordinated in a common hexadentate fashion, whereas the other one is coordinated only through a carboxylate function(s). Such a coordination is weak (see Table 1 for the corresponding log *K* values) but coordination of the second ligand molecule is evidenced by the changes of the NMR spectra: in the acidic solution, higher chemical shift of the complex ^45^Sc NMR signal points to a coordination of negatively charged carboxylate group in [Sc­(L)­(HL)] replacing the uncharged water molecules present in the 1:1 complex. When the pH is further increased, the species [Sc­(L)­(OH)]/[Sc_2_(L)_2_(μ-OH)_2_] are formed with release of the free ligand whose signals can be observed in the ^1^H NMR spectra. On the other hand, the signals of the free ligand are also present in acidic solutions, but they are broader than in the case of a pure ligand solution. The broadening points to a fast exchange between free ligand molecules and the carboxylate-bound protonated ligand molecule in the [Sc­(L)­(HL)] species. Such dynamics also broaden signals of hexadentately coordinated ligand in the 2:1 species.

The agreement of species abundance in the NMR spectra with the distribution diagrams shown above confirms the chemical model selected for the potentiometric data evaluation.

Chemical shifts of the observed signals together with their half-widths are given in Table 3.

**Table 3:** ^45^Sc NMR Signals of Individual Sc^III^ Complexes in the Studied Systems (*c*_Sc_ = 25 mM, 25 °C)

	H_3_nota		H_2_ **L^1^**		H_2_ **L^2^**	
species^a^	δ [ppm]	*w*_1/2_ [Hz]	δ [ppm]	*w*_1/2_ [Hz]	δ [ppm]	*w*_1/2_ [Hz]
Sc^III^ aqua ion	7	∼60	8	∼130	7	∼130
[Sc(L)]	∼85	∼1700	76	∼1770	75	∼1880
[Sc(L)(OH)]	∼145	∼2600	∼140	∼2600	∼147	∼3200
[Sc_2_(L)_2_(OH)_2_]	∼90	∼500	∼89	∼670	∼90	∼670
[Sc(L)(HL)]	∼110	∼2000	∼106	∼5600	∼107	∼5700

a Charges are omitted.

### Solid-State Structures of the Ternary Complexes

The Sc^III^ ion has a coordination number of 8 in the solid-state structures of the studied complexes (see above). Six coordination sites are occupied by the macrocyclic ligand, and the remaining two positions are occupied by water molecules or other coordinating species. In the course of crystallization attempts, single crystals of the ternary complex Li_2_[{Sc­(nota)}_2_(O_2_)]·10H_2_O (Figure 6) were grown from solution accidentally contaminated with hydrogen peroxide. The ongoing experiments showed that the ternary complex forms and crystallizes reproducibly upon peroxide addition and single crystals of ternary complexes with peroxide anion were also grown for H_2_ **L** ^1^ (Figure S14) and H_2_ **L** ^2^ (Figure S15). In all structures, the Sc^III^ ion shows similar geometry. The Sc^III^ ion is octacoordinated with the ligand and both oxygen atoms of (μ-η:^2^η^2^-O_2_)^2–^ anion bridging two symmetrical {Sc­(L)} units. Coordination spheres are analogous to those described for the binary complexes. The N_3_O_4_O_1_ coordination spheres exhibit similar geometries and coordination distances as above (Table S1); the only difference lies in the position of the apical oxygen atom, which is not capping a center of the O_4_ plane due to the shortness of the O–O bond in the peroxido ligand and, thus, the Sc–O_apical_ vector is inclined with respect to the O_4_ plane.

**Figure 6:**
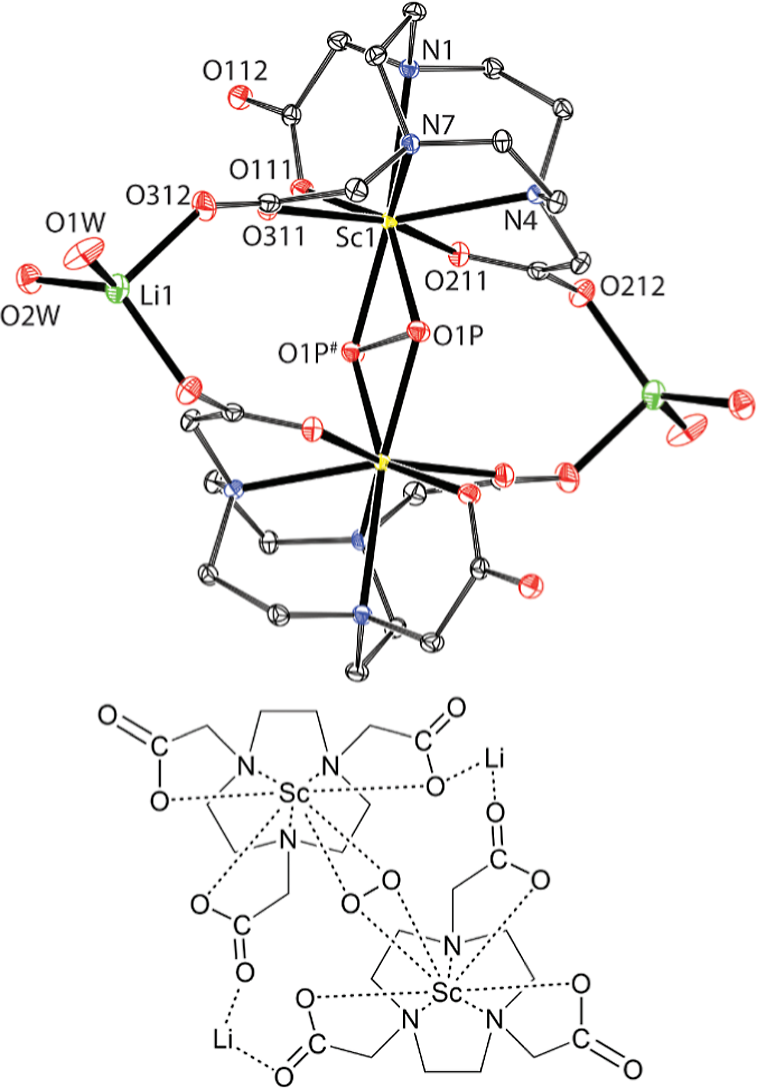
Molecular structure of dimeric species [Li­(H_2_O)_2_]_2_[{Sc­(nota)}_2_(O_2_)] found in the crystal structure of Li_2_[{Sc­(nota)}_2_(O_2_)]·10H_2_O. The hydrogen atoms and charges are not shown for clarity.

The bridging coordination of peroxide is quite common, and a number of solid-state structures of complexes with rare-earth-metal ions have been reported.^38,39^ The reported complexes are mostly mixed lanthanide­(III) complexes bearing peroxide anion and various ligands including, e.g., carbonate, carboxylate, amide, chloride, pyridine, or various polydentate ligands. Peroxide anion is mostly coordinated in a similar geometry as in the presented complexes, bridging two or even three metal ions.

The peroxide-containing structures showed that water molecules coordinated in solution can be replaced with other coordinating species. Such substitution might change in the complex charge, size, and hydrophilicity which are important for in vivo behavior. Thus, we investigated the interactions of the studied Sc^III^ complexes with oxalate, which was chosen as a naturally occurring bidentate ligand.

Single crystals of two ternary complexes, Li_6_[{Sc­(nota)}_3_(C_2_O_4_)_3_]·14H_2_O (Figure 7) and [{Sc­(**L** **^1^**)}_2_(C_2_O_4_)]·10H_2_O (Figure 8), were grown. Both complexes show geometries of the coordination polyhedron similar to those discussed above. The N_3_O_4_O_1_ coordination sphere is formed again, and the oxalate anions occupy the apical position and one position in the O_4_ plane. Oxalate anions are coordinated in a μ-κ^2^-O,O‴(Sc)-κ^2^-O′O″ mode bridging the {Sc­(nota)} unit with Li^I^ ion or two {Sc­(**L** ^1^)} units, respectively. The Sc–O distance to the oxalate oxygen atom in the apical position is slightly longer if compared to oxygen atoms bound in the equatorial position.

**Figure 7:**
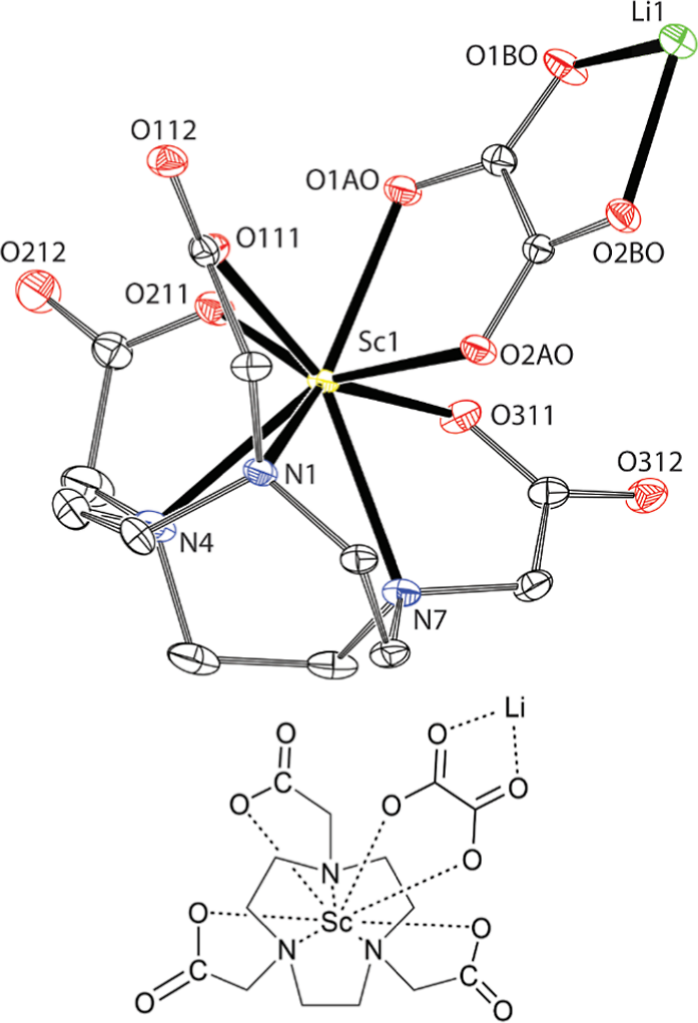
Molecular structure of the selected [Li­{Sc­(nota)}­(C_2_O_4_)]^−^ fragment found in the crystal structure of Li_6_[{Sc­(nota)}_3_(C_2_O_4_)_3_]·14H_2_O. The hydrogen atoms and charges are not shown for clarity.

**Figure 8:**
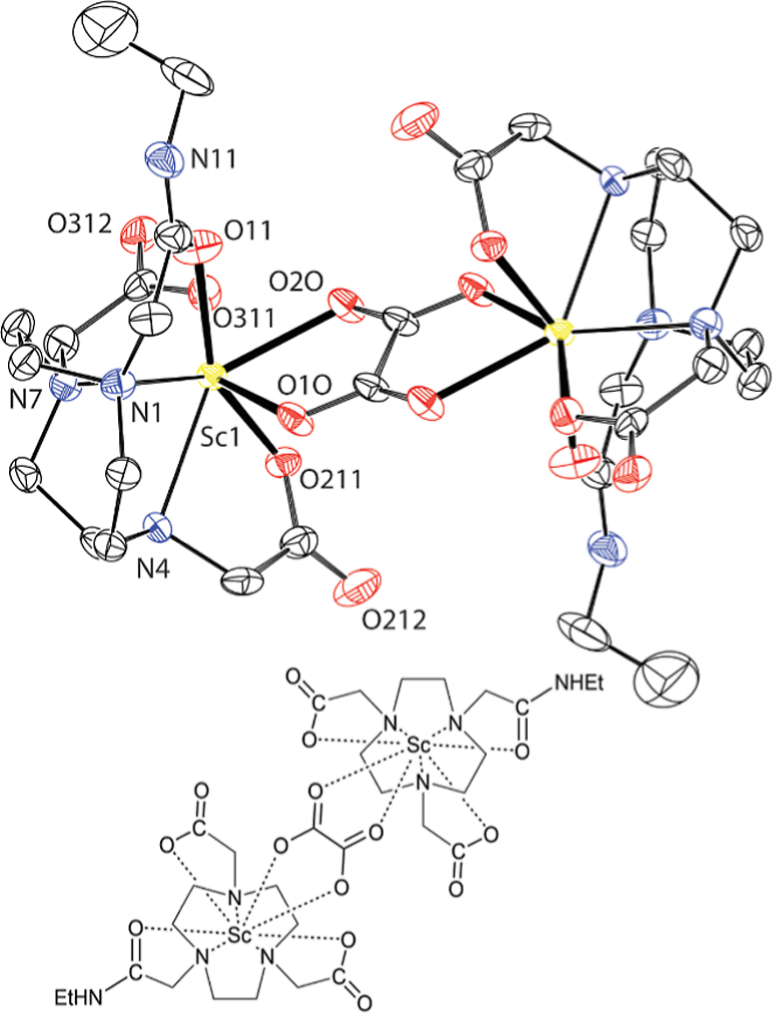
Molecular structure of dimeric species [{Sc­(**L** ^1^)}_2_(C_2_O_4_)] found in the crystal structure of [{Sc­(**L** ^1^)}_2_(C_2_O_4_)]·10H_2_O. The more abundant position of the disordered macrocycle is shown. The hydrogen atoms and charges are not shown for clarity.

All determined solid-state structures show similar geometries of Sc^III^ coordination polyhedra, and a similar geometry was also previously reported for [Sc­(nota)­(OAc)].^2^ It indicates that the Sc^III^ complexes with nota-like ligands prefer octacoordination with the N_3_O_4_O_1_ geometry. Such geometry is significantly different from N_4_O_4_ geometry of Sc^III^ complexes with dota-like ligands which are typically used as universal carriers of metal radionuclides.^1,40,41^ However, coordination distances to macrocyclic nitrogen and pendant oxygen atoms are similar in complexes of dota-like and nota-like ligands. Different geometry was also reported for Sc^III^ complexes with 1,4,7-triazacyclononane derivatives bearing phenolate pendant arms, in which the ligand is hexacoordinated in the octahedral geometry and Sc–O distances are by ∼0.2 Å shorter than those found in the Sc^III^ complexes with H_3_nota and its amides.^42,43^ Similarly, octahedral coordination geometry was reported in the solid-state structures of H_3_nota complexes with Co^III^, Fe^III^, and Ga^III^ ions.^44–46^ The ionic diameters of these metal ions are smaller than that of the Sc^III^ ion and, thus, coordination bond lengths in the Sc^III^ complexes are the longest among these complexes. The important difference between complexes with octahedral and N_3_O_4_O_1_ geometry is the rotation of the pendant arms. All three pendant arms in octahedral complexes show the same direction of rotation, forming a structure close to *C*_3_ symmetry. On the contrary, the N_3_O_4_O_1_ geometry requires two pendant arms to be rotated in opposite directions to form the space for the additional ligand to form the O_4_ plane. The rotation of the remaining pendant arm is negligible, forming a structure close to the *C*_s_ geometry.

The N_3_O_4_O_1_ geometry was also previously reported for the Sc^III^ complex with H_4_aazta,^32^ and a similar geometry was predicted also by DFT calculations for the Sc^III^ complex with H_3_mpatcn.^4,5^ Geometry of the N_3_O_4_O_1_ polyhedron is similar in all reported structures (Table S2) Sc^III^ ion is placed between N_3_ and O_4_ planes, closer to the O_4_ plane (*d*(Sc···N_3_) = 1.7–1.8 Å, *d*(Sc···O_4_) = 0.5–0.7 Å). The distance between the Sc^III^ ion and the apical oxygen atom is somewhat shortened in the structures with a coordinated peroxide anion (*d*(Sc–O) = 2.12 Å) in comparison with other described complexes (*d*(Sc–O) = 2.25–2.44 Å).

### Solution NMR Study of the Ternary Complexes

To understand the behavior of the ternary complexes in solution, the systems of the title Sc^III^ complexes with oxalate were also studied by ^45^Sc, ^1^H, and ^13^C NMR. Figures 9 and S16–S18 show spectra of samples with a constant concentration of oxalate anion and variable concentration of the Sc^III^ complexes. In the Sc^III^–H_3_nota–oxalate system, the interaction with oxalate anion leads to the ^45^Sc NMR signal at ∼75 ppm with a significantly smaller half-width than that of the free [Sc­(nota)] complex. The complex shows a decreased symmetry and a rigidified structure as ^1^H NMR signals are split, and the splitting is clearly observed. In addition, a broad signal at ∼50 ppm appears under the excess oxalate. This signal might be ascribed to the species, in which two oxalate anions are bound to the Sc^III^ ion in a monodentate mode or replace one or two pendant arms in the metal ion coordination sphere. However, the coordination of oxalate likely has a dynamic character as no ^13^C NMR signal of free oxalate anion was found in the spectra when oxalate was present in the excess. Contrary, with increasing complex-to-oxalate ratio, a broad signal at 170 ppm gradually appears (Figure S16). Similarly, under the [Sc­(nota)] complex excess, an additional ^45^Sc NMR signal at ∼90 ppm appears. These signals might correspond to the species in which one oxalate binds two Sc^III^ complexes, similar to the situation observed in the solid state (see above). The dinuclear species does not form quantitatively, pointing to weak coordination of the second complex unit. The formation of this species with 2:1 complex-to-oxalate ratio is also supported by ^1^H NMR spectra showing significant broadening of the signals at the complex excess, whereas neither signals of the free complex nor signals of the 1:1 ternary species were observed (Figure S16).

**Figure 9:**
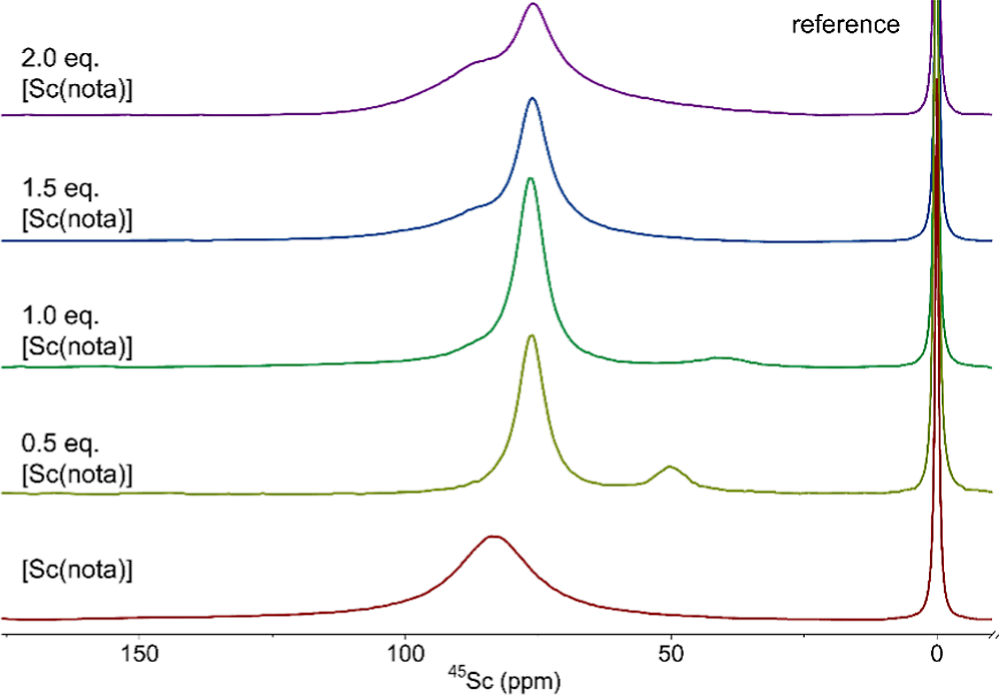
^45^Sc NMR spectra of the Sc^III^–H_3_nota–oxalate system as a function of the complex/oxalate ratio (*c*_oxalate_ = 25 mM, pH 7.0, imidazole/HCl, *c*_buffer_ = 0.25 M).

The same behavior was observed for both amide ligands. Remarkable is the splitting of the ^1^H NMR signals, resulting from the decreased symmetry of the amide ligand in comparison with the parent H_3_nota. However, the detailed view of ^1^H NMR signals (nicely observable for the methyl group of the ethylamide) in the Sc^III^–H_2_ **L** ^1^–oxalate system showed that not one but two species are present (Figure 10). The relative intensities of the two signals do not change with the complex-to-oxalate ratio, which indicates the same stoichiometry of both species. To explain this observation, one must consider the geometry of the coordination sphere. The solid-state structures (see above) showed that the coordination sphere has N_3_O_4_O_1_ arrangement; the N_3_ and O_4_ planes are coplanar. One oxalate oxygen atom is coordinated in the apical position, whereas the other one completes the O_4_ plane. However, there are two possible locations of the oxalate oxygen atom in the O_4_ plane: between two carboxylates or between the amide and carboxylate pendants. Simultaneous presence of the two isomers explains the presence of these two methyl signals in the ^1^H NMR spectra. The two species could not be distinguished in ^45^Sc NMR due to the large width of the signal and the same geometry of the coordination sphere in both species. To support the explanation, the theoretical structures and energies of both isomers were calculated (Figure S19). The calculated structures are in very good agreement with those found in the solid state. The more stable isomer has oxalate bound between the carboxylate and amide pendants, likely due to the electrostatic repulsion between negatively charged oxalate and carboxylates. However, isomers’ energies differ only by 1.5 kcal/mol (free energies in the implicit solvent at 25 °C), which confirms their simultaneous presence in solution. The observed isomerism is indirect proof that the N_3_O_4_O_1_ coordination sphere is maintained in the solution.

**Figure 10:**
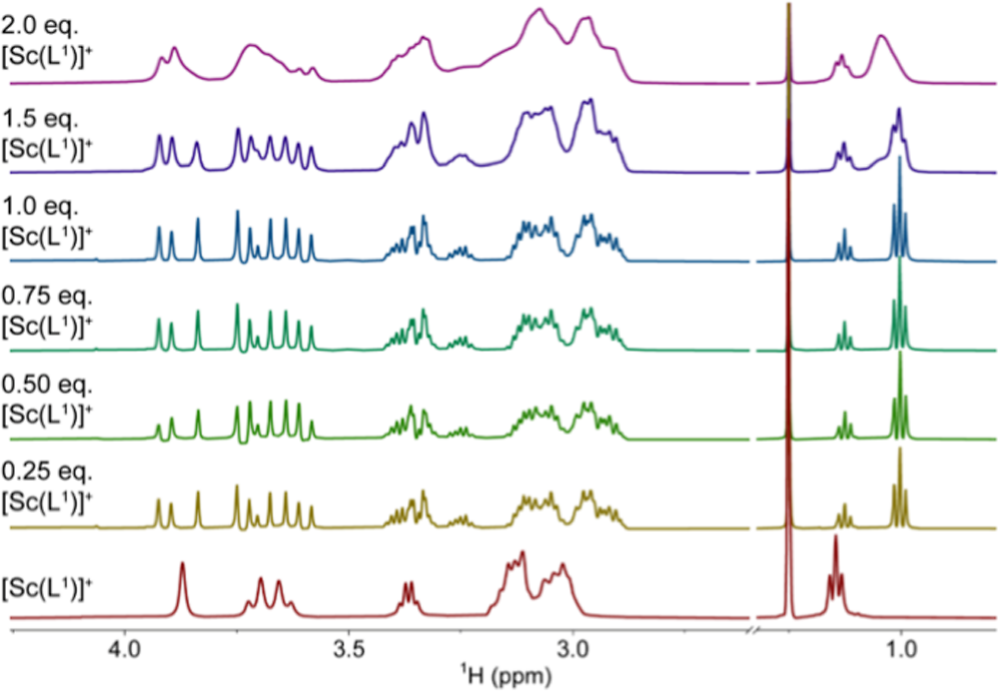
^1^H spectra of the Sc^III^–H_2_ **L** ^1^–oxalate system as a function of the complex/oxalate ratio (*c*_oxalate_ = 25 mM, pH 7.0, imidazole/HCl, *c*_buffer_ = 0.25 M).

It is necessary to mention that Sc^III^ complexes with the amide ligands, H_2_ **L** ^1^ and H_2_ **L** ^2^, show limited stability. They slowly decompose in strongly alkaline solutions or under excess oxalate, yielding insoluble Sc^III^ oxalate or hydroxide.

## Conclusions

H_3_nota and its monoamides form stable Sc^III^ complexes, in which the Sc^III^ ion is octacoordinated, possessing N_3_O_4_O_1_ geometry. The ligand occupies six positions of the coordination polyhedron. In the solution, the remaining two positions are occupied by water molecules or by the second ligand molecule if present in excess. Consequently, carboxylate-bridged oligomers were found in the solid state.

The water molecules are also easily replaced with other coordinating ligands, giving rise to ternary complexes. In the solid state, the ternary complexes with peroxido or oxalato ligands show a similar N_3_O_4_O_1_ geometry as the binary complexes. Multinuclear NMR measurements demonstrated that the ternary complexes are also present in solution. The ^45^Sc NMR was found to be a suitable tool for investigation of these systems, as species differing in the ligand-to-metal ratio show individual signals differing significantly in their chemical shift. In addition, two isomeric forms of the ternary complex were found in the case of nota-monoamides due to their asymmetry, leading to two possible positions of the coordinated oxalate anion in the coordination polyhedron.

It is necessary to mention that the unclosed coordination sphere of Sc^III^ complexes with H_3_nota and its derivatives bearing monodentate pendant arms disfavors their use in nuclear medicine. The unclosed coordination sphere decreases kinetic inertness of the complexes, which is a crucial parameter of the metal-based radiopharmaceuticals. However, the presented results offer a possible improvement. The fact that N_3_O_4_O_1_ geometry was found in all structures indicates that this is the preferred geometry in the Sc^III^ complexes with nota-like ligands. This finding shows that increasing ligand denticity by introduction of bidentate or tridentate pendant arms on the tacn core might be a promising strategy for development of ligands for the effective complexation of Sc^III^ radioisotopes.

## Supplementary Material



## Notes

NMR spectra of binary and ternary complexes, distribution diagrams of studied systems, overall stability constants, solid-state structures of complexes, crystallographic tables, and calculated structures of isomers (PDF)
