# Supplementary material for: Sc(III) Complexes of 1,4,7-Triazacyclononane-1,4,7-triacetic Acid and Its Monoamides
Source: Inorg Chem. 2025 Nov 12;64(46):22928–38. doi: 10.1021/acs.inorgchem.5c04142 (PMC12648657; doi:10.1021/acs.inorgchem.5c04142)
Supplement: Supplementary file 1 [file ic5c04142_si_001.pdf]

Supporting information

Sc(III) complexes of 1,4,7-triazacyclononane-1,4,7-triacetic acid and its monoamides

Jan Kubinec,[a] Filip Koucký,[a] Adam Svítok,[a] Jan Faltejsek,[a,b] Jan Kotek,[a] Vojtěch Kubíček [a]* and Petr Hermann [a]

[a] Charles University, Faculty of Science, Department of Inorganic Chemistry, Hlavova 8, 128 43 Prague 2, Czech Republic; email: kubicek@natur.cuni.cz

[b] Czech Academy of Sciences, Institute of Organic Chemistry and Biochemistry, Flemingovo náměstí 542/2, 160 00 Prague 6, Czech Republic

Table of contents

Figure S1. ^{1}H, $^{13}C\{^{1}H\}$ and ^{45}Sc NMR spectra of Sc^{III}–H_3nota complex

Figure S2. ^{1}H, $^{13}C\{^{1}H\}$ and ^{45}Sc NMR spectra of Sc^{III}–$H_2\mathbf{L^1}$ complex

Figure S3. ^{1}H, $^{13}C\{^{1}H\}$ and ^{45}Sc NMR spectra of Sc^{III}–$H_2\mathbf{L^2}$ complex

Figure S4. ^{1}H, ^{13}C and ^{45}Sc NMR spectra of the ternary system Sc^{III}–H_3nota–oxalate

Figure S5. ^{1}H, ^{13}C and ^{45}Sc NMR spectra of the ternary system Sc^{III}–$H_2\mathbf{L^1}$–oxalate

Figure S6. ^{1}H, ^{13}C and ^{45}Sc NMR spectra of the ternary system Sc^{III}–$H_2\mathbf{L^2}$–oxalate

Figure S7. The disorder found in the crystal structure of $[\{Sc(\mathbf{L^1})\}_2(C_2O_4)]\cdot 10H_2O$.

Table S1. Coordination bond lengths (Å) in the discussed Sc^{III} complexes

Table S2. Parameters of the coordination polyhedra (Å) in the discussed Sc^{III} complexes

Table S3. Experimental crystallographic data of Sc^{III} complexes

Figure S8. Examples of experimental potentiometric data and corresponding fits of Sc^{III}– H_3nota system

Figure S9. Examples of experimental potentiometric data and corresponding fits of Sc^{III}– $H_2\mathbf{L^1}$ system

Table S4. Overall stability constants of Sc^{III} complex with the studied ligands

Figure S10. ^{45}Sc and ^{1}H NMR spectra of the Sc^{III}–$H_2\mathbf{L^1}$ system as a function of pH

Figure S11. ^{45}Sc and ^{1}H NMR spectra of the Sc^{III}–$H_2\mathbf{L^1}$ system as a function of pH

Figure S12. ^{45}Sc and ^{1}H NMR spectra of the Sc^{III}–$H_2\mathbf{L^2}$ system as a function of pH

Figure S13. ^{45}Sc and ^{1}H NMR spectra of the Sc^{III}–$H_2\mathbf{L^2}$ system as a function of pH

Figure S14. The molecular structure of dimeric species $[(Sc(\mathbf{L^1}))_2(O_2)]$ found in the crystal structure of $[\{Sc(\mathbf{L^1})\}_2(O_2)]\cdot 10H_2O$

Figure S15. The molecular structure of dimeric species $[\{Sc(\mathbf{L^2})\}_2(O_2)]$ found in the crystal structure of $[\{Sc(\mathbf{L^2})\}_2(O_2)]\cdot 12H_2O$

Figure S16. ^{45}Sc, ^{1}H and $^{13}C\{^{1}H\}$ NMR spectra of Sc^{III}-H_3nota-oxalate system as function of complex:oxalate ratio

Figure S17. ^{45}Sc, ^{1}H and $^{13}C\{^{1}H\}$ NMR spectra of Sc^{III}-$H_2\mathbf{L^1}$-oxalate system as function of complex:oxalate ratio

Figure S18. ^{45}Sc, ^{1}H and $^{13}C\{^{1}H\}$ NMR spectra of Sc^{III}-$H_2\mathbf{L^2}$-oxalate system as function of complex:oxalate ratio

Figure S19. Calculated structures of two isomers of the ternary complex $[Sc(\mathbf{L^1})(ox)]^-$

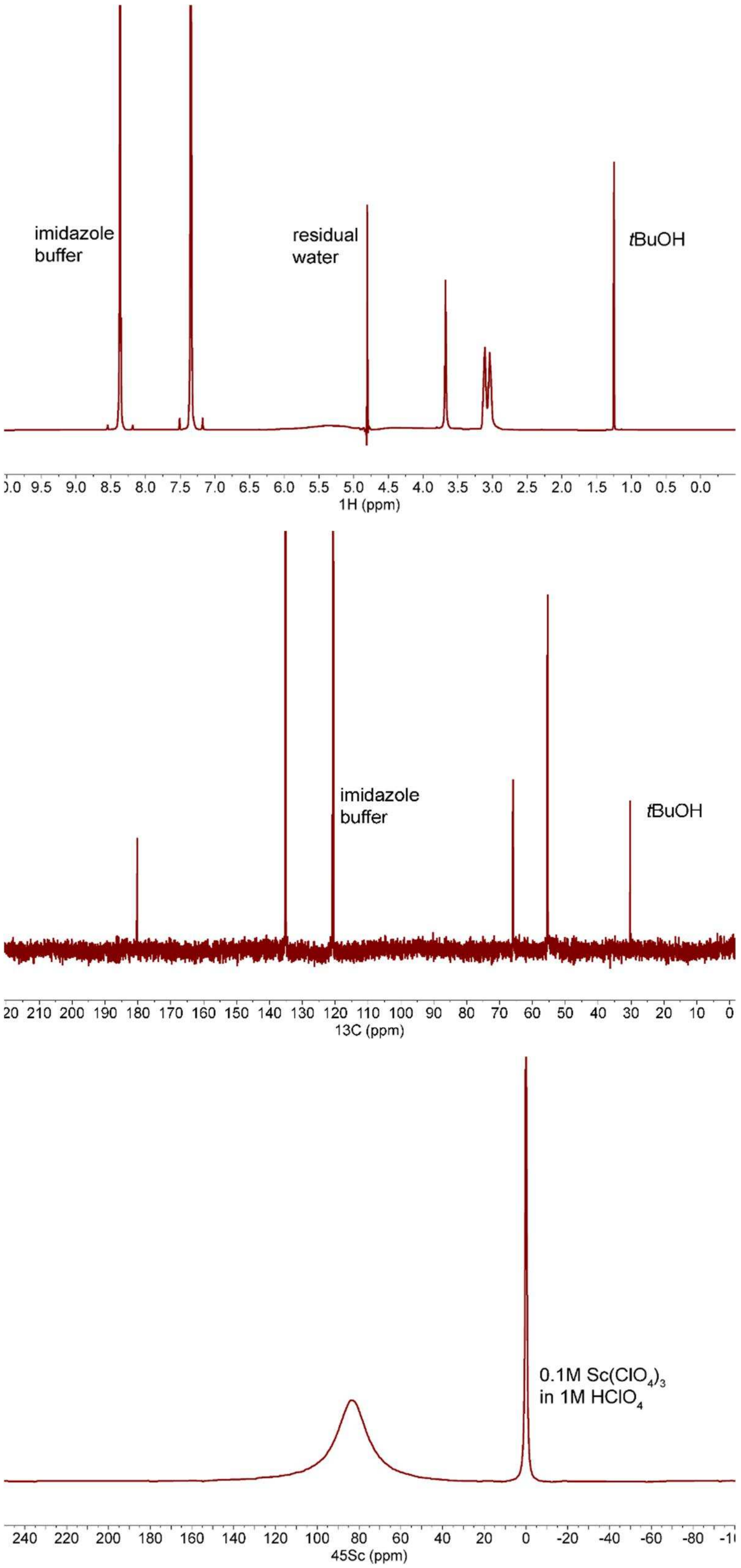

Figure S1. ^{1}H, ^{13}C{^{1}H} and ^{45}Sc NMR spectra of the Sc^{III}–H$_3$nota complex at pH 7.0 (in D$_2$O, 400 MHz).

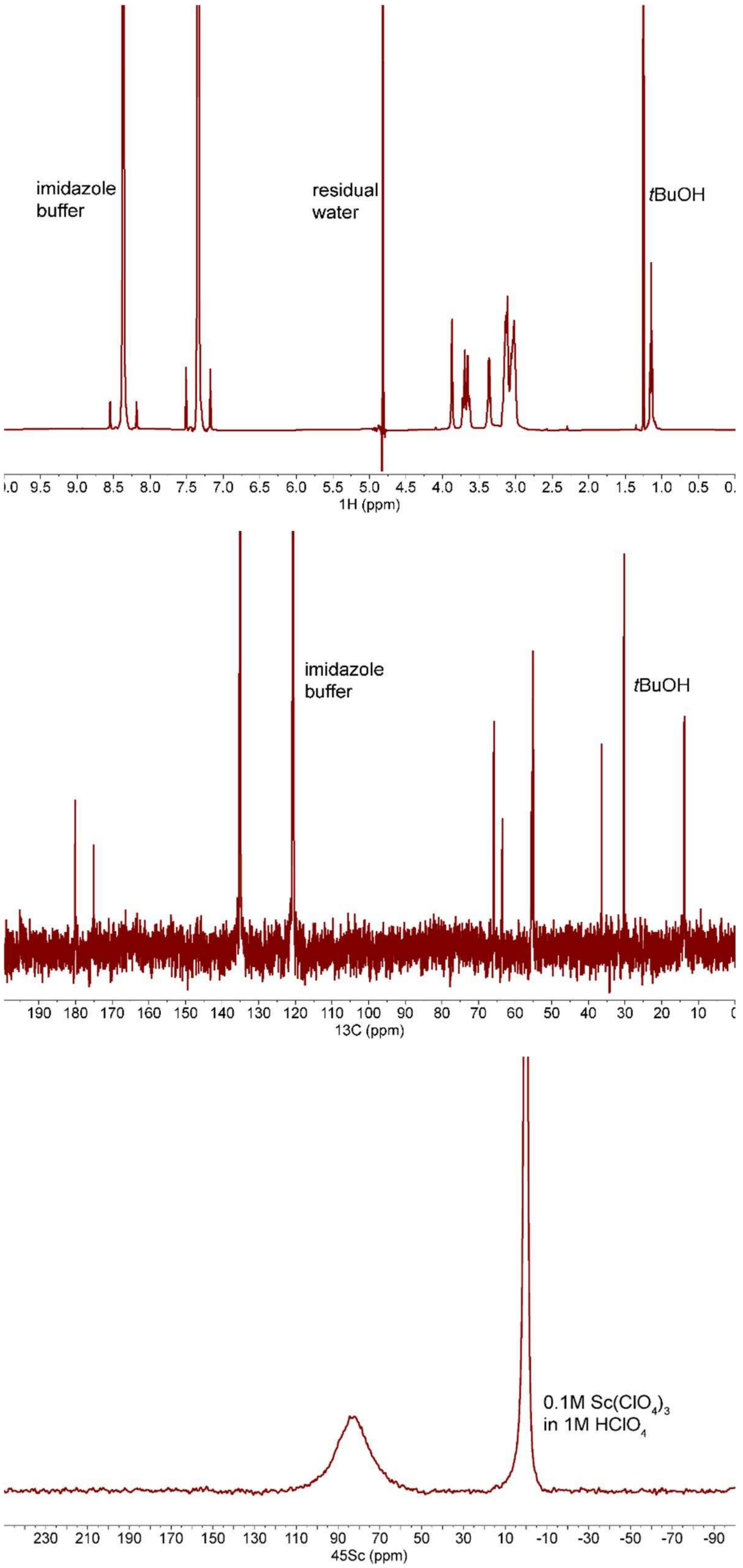

Figure S2. ^{1}H, ^{13}C{^{1}H} and ^{45}Sc NMR spectra of the Sc^{III}–H$_2$**L**^{1} complex at pH 7.0 (in D$_2$O, 400 MHz).

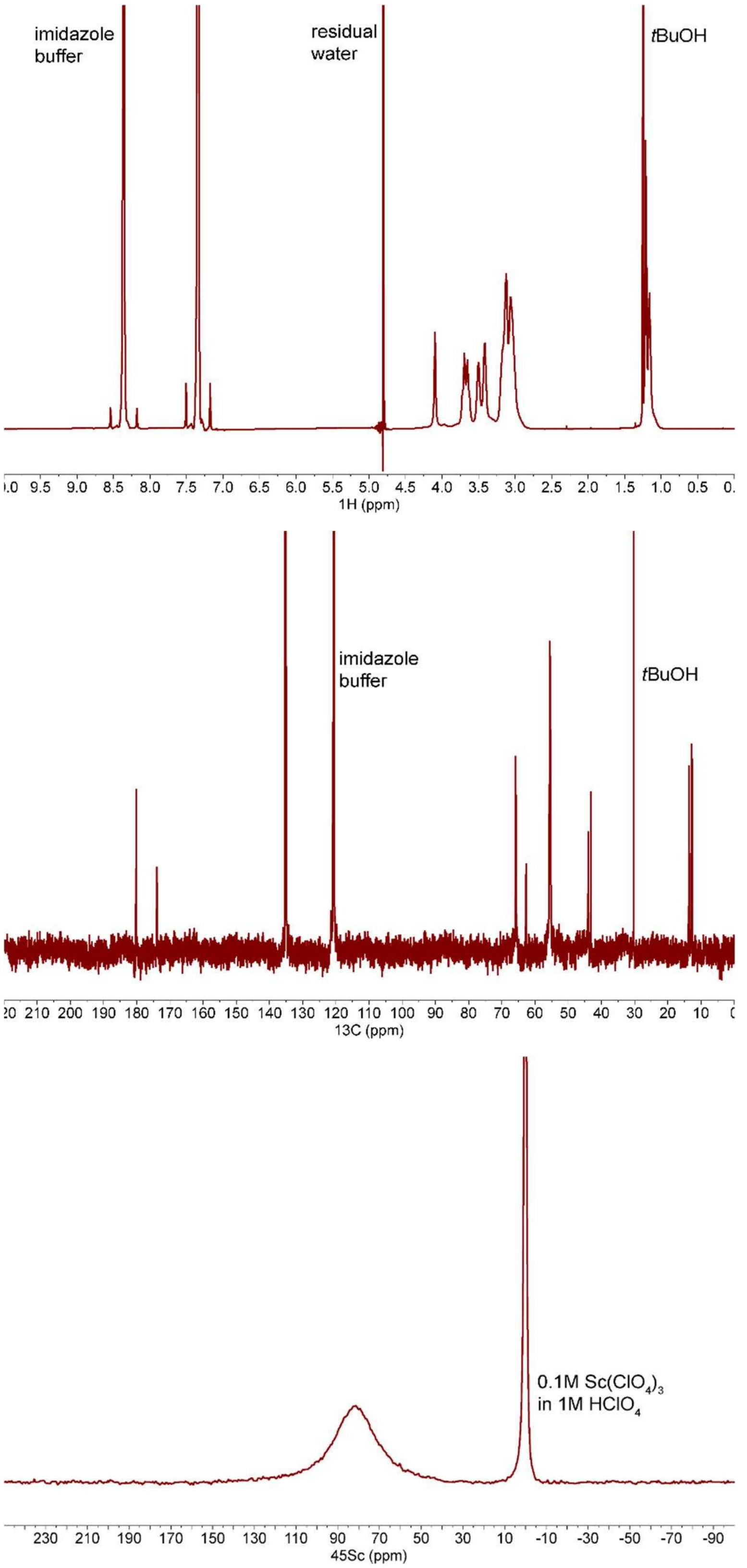

Figure S3. ^{1}H, $^{13}C\{^{1}H\}$ and ^{45}Sc NMR spectra of the Sc^{III}–H_2**L^2** complex at pH 7.0 (in D_2O, 400 MHz).

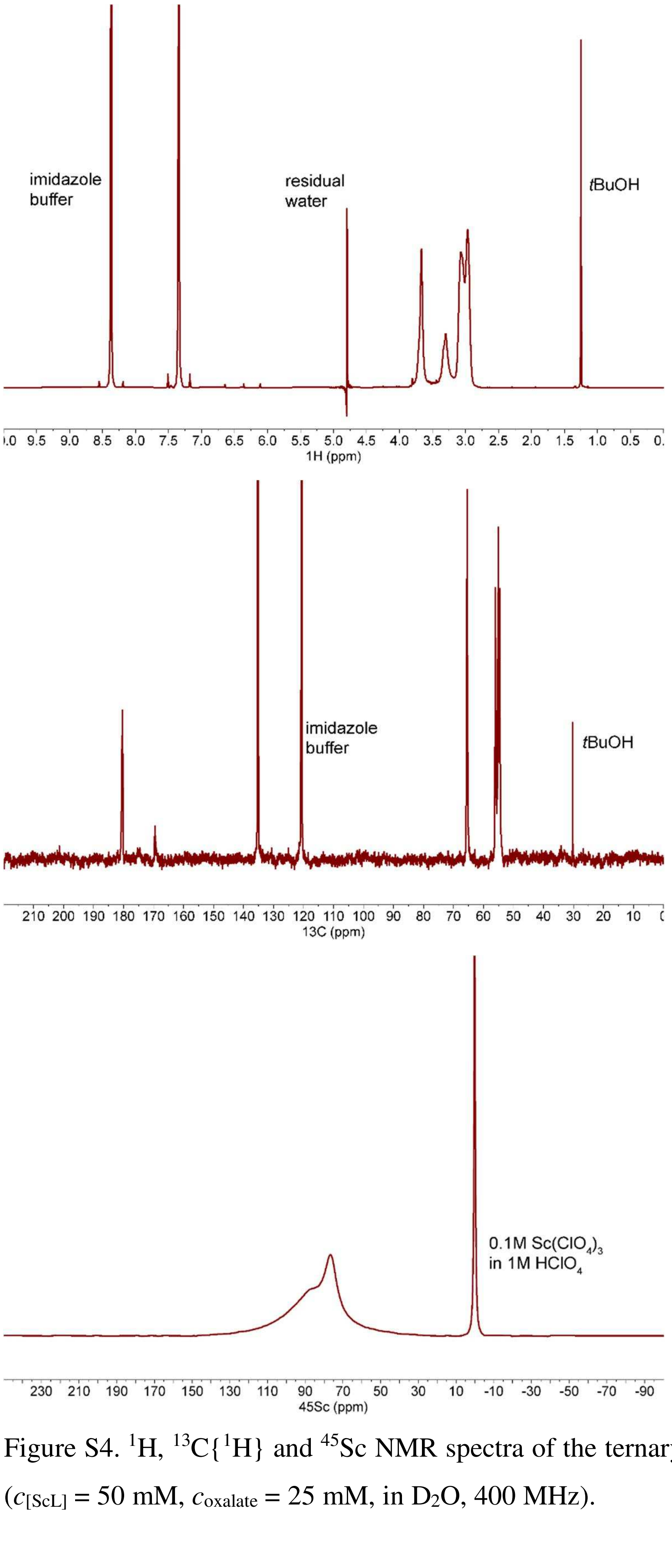

Figure S4. ^{1}H, ^{13}C{^{1}H} and ^{45}Sc NMR spectra of the ternary system Sc^{III}–H$_3$nota–oxalate at pH 7.0 ($c_{[ScL]}$ = 50 mM, $c_{oxalate}$ = 25 mM, in D_2O, 400 MHz).

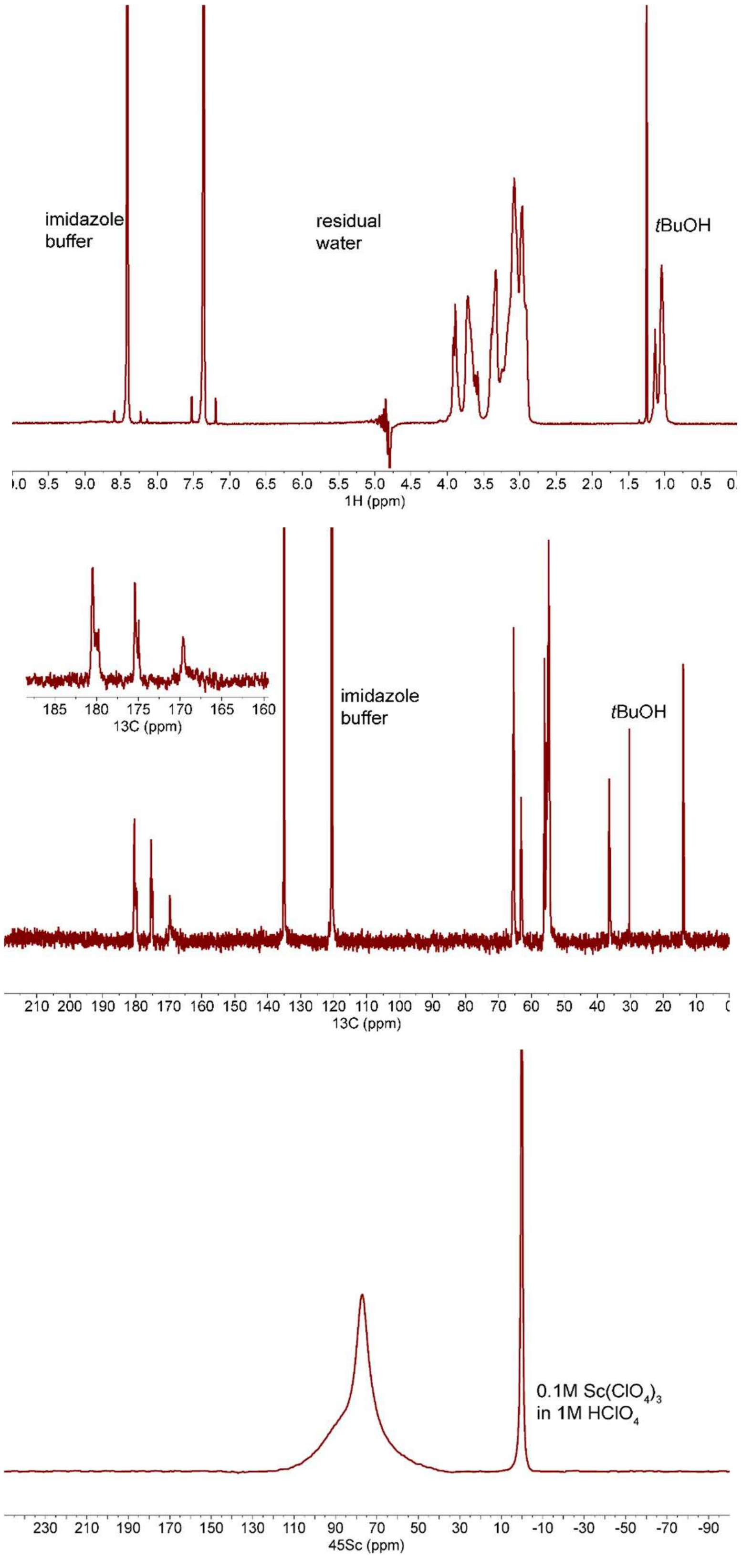

Figure S5. ^{1}H, $^{13}C\{^{1}H\}$ and ^{45}Sc NMR spectra of the ternary system Sc^{III}–$H_2\mathbf{L^1}$–oxalate at pH 7.0 ($c_{[ScL]}$ = 50 mM, $c_{oxalate}$ = 25 mM, in D_2O, 400 MHz).

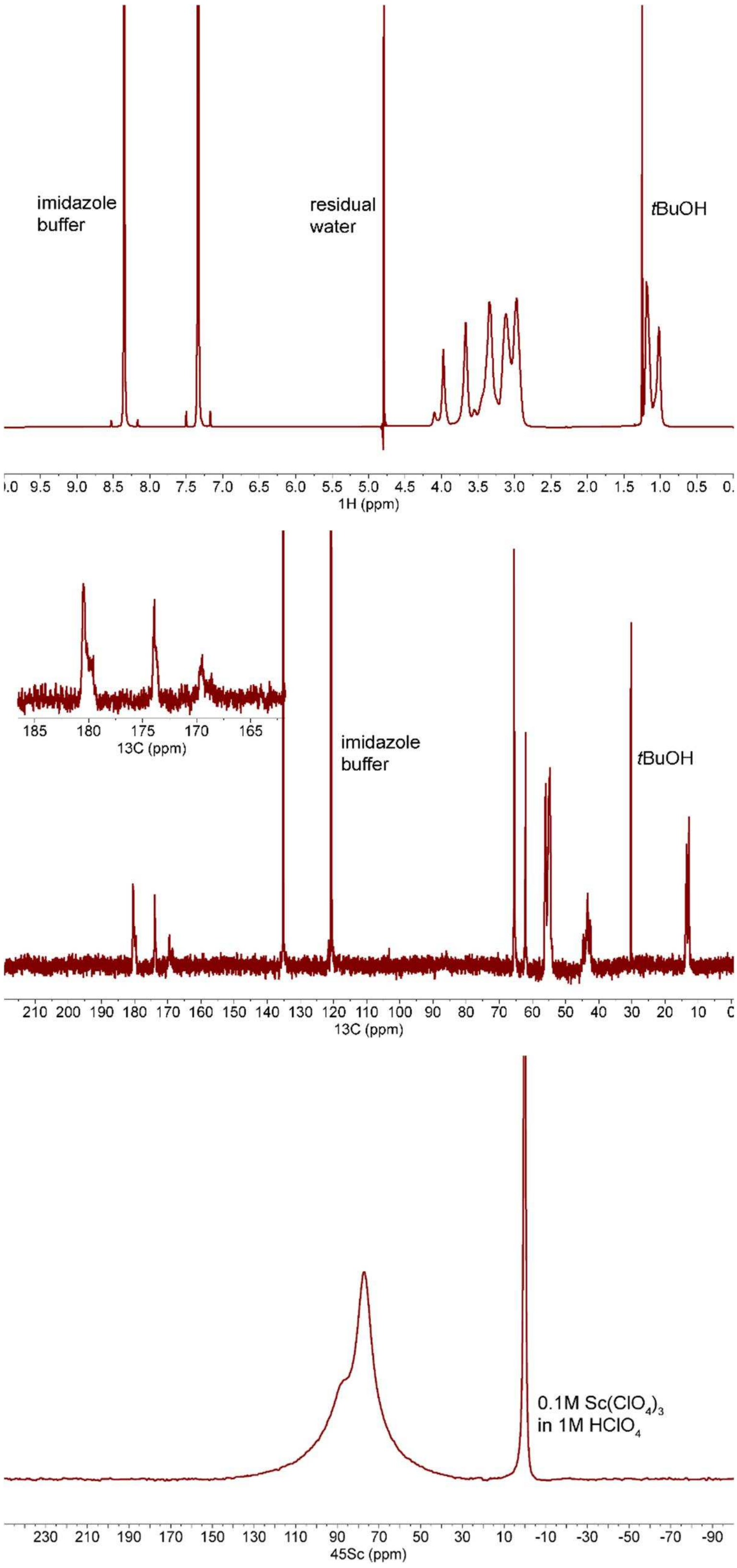

Figure S6. ^{1}H, $^{13}C\{^{1}H\}$ and ^{45}Sc NMR spectra of the ternary system Sc^{III}–$H_2\mathbf{L^2}$–oxalate at pH 7.0 ($c_{[ScL]}$ = 50 mM, $c_{oxalate}$ = 25 mM, in D_2O, 400 MHz).

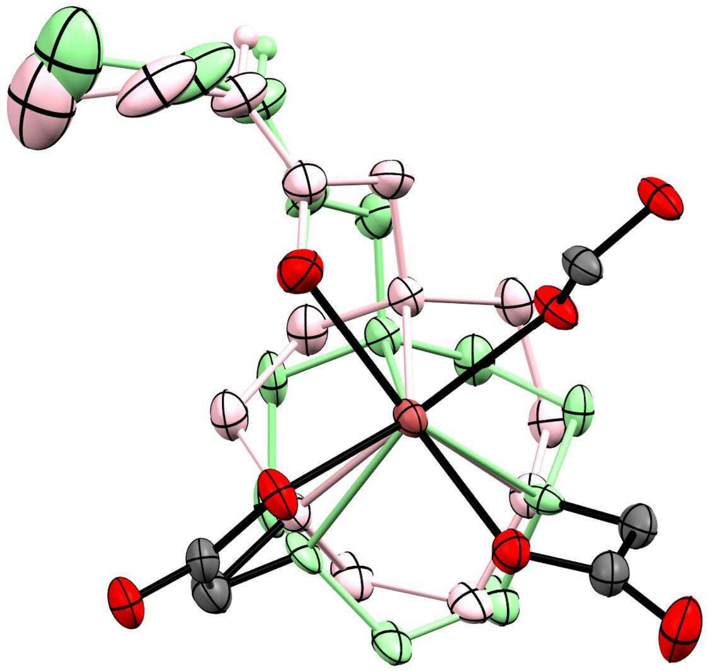

Figure S7. The disorder found in the crystal structure of [{Sc(**L**[1])}$_2$(C_2O_4)]·10H_2O. The structurally independent part of the complex is shown. The non-disordered atoms are shown in brown (Sc), red (O) and grey (C), and are connected with the black bonds. The disordered parts are shown in pink and greenish. The coordinated CO_2 fragment in the right top corner corresponds to half of the centrosymmetric oxalate ligand.

Table S1. Coordination bond lengths (Å) in the discussed Sc^{III} complexes

	[Sc{Sc(nota)}$_6$] $Cl_3\cdot 26H_2O$	Li_3[{Sc(**L**^{1})}$_6$]$_2$ $(ClO_4)_{10}Cl_5$ $\cdot 6Me_2CO\cdot 35.5H_2O$ [d]	Li_2[{Sc(nota)}$_2$(O_2)] $\cdot 10H_2O$	[{Sc(**L**^{1})}$_2$(O_2)] $\cdot 10H_2O$	[{Sc(**L**^{2})}$_2$(O_2)] $\cdot 12H_2O$	Li_6[{Sc(nota)}$_3$(C_2O_4)$_3$] $\cdot 14H_2O$ [g]	[{Sc(**L**^{1})}$_2$(C_2O_4)] $\cdot 10H_2O$	[Sc(nota) (AcO)] [1]
Sc1–N1	2.358(2)	2.41–2.45	2.445(1)	2.389(1)	2.439(1)	2.43–2.46	2.421(2)/2.484(6)[i]	2.384
Sc1–N4	2.376(2)	2.34–2.47	2.363(1)	2.448(1)	2.380(1)	2.43–2.44	2.462(10)/2.546(10)[i]	2.374
Sc1–N7	2.387(2)	2.37–2.48	2.424(1)	2.434(1)	2.437(1)	2.40–2.44	2.374(4)/2.449(26)[i]	2.393
Sc1–O11	2.102(1)	2.15–2.25	2.121(1)	2.243(1)	2.140(1)	2.14–2.18	2.195(1)	2.116
Sc1–O21	2.216(1)	2.12–2.15	2.244(1)	2.169(1)	2.174(1)	2.11–2.18	2.128(1)	2.165
Sc1–O31	2.126(2)	2.13–2.18	2.218(1)	2.134(1)	2.199(1)	2.16–2.21	2.106(1)	2.155
Sc1–OX[a]	2.205(1)[c]	2.11–2.16[c]	2.066(1)[f]	2.064(1)[f]	2.058(1)[f]	2.12–2.15[h]	2.175(1)[h]	2.213[j]
Sc1–OY[b]	2.442(1)[c]	2.26–2.31[e]	2.124(1)[f]	2.127(1)[f]	2.127(1)[f]	2.25–2.28[h]	2.312(1)[h]	2.377[j]

[a] Oxygen atom in the O_4 plane; [b] Oxygen atom in the apical position; [c] Carboxylate of the neighbouring complex unit; [d] Six structurally independent {Sc(L)} units; [e] Water molecule; [f] Peroxide; [g] Three structurally independent {Sc(L)} units; [h] Oxalate; [i] Two disordered positions; [j] Acetate.

[1] K. E. Aldrich, I. A. Popov, H. D. Root, E. R. Batista, S. M. Greer, S. A. Kozimor, L. M. Lilley, M. Y. Livshits, V. Mocko, M. T. Janicke, B. L. Scott, B. W. Stein, P. Yang; Synthesis, solid-state, solution, and theoretical characterization of an "in-cage" scandium-NOTA complex. *Dalton Trans.* **2022**, *51*, 9994–10005.

Table S2. Parameters of the coordination polyhedra (Å) in the discussed Sc^{III} complexes

	[Sc{Sc(nota)}$_6$] Cl_3·26H_2O	Li_3[{Sc(**L**^{1})}$_6$]$_2$ $(ClO_4)_{10}Cl_5$ ·6Me_2CO ·35.5H_2O [e]	Li_2[{Sc(nota)}$_2$(O_2)] ·10H_2O	[{Sc(**L**^{1})}$_2$(O_2)] ·10H_2O	[{Sc(**L**^{2})}$_2$(O_2)] ·12H_2O	Li_6[{Sc(nota)}$_3$ $(C_2O_4)_3$]·14H_2O	[{Sc(**L**^{1})}$_2$ (C_2O_4)]·10H_2O	[Sc(nota) (AcO)] [1]	M[Sc(aazta)(H_2O)] ·4H_2O [a 2]
d(Sc–N_3)	1.72	1.81–1.82	1.77	1.79	1.78	1.80	1.78	1.72–1.75	1.83
d(Sc–O_4)	0.68	0.52–0.57	0.63	0.64	0.63	0.57–0.62	0.59	0.68–0.76	0.50
d(Sc–O)	2.44	2.24–2.31	2.12	2.13	2.13	2.25–2.28	2.31	2.32–2.38	2.25

[a] Composition {[$(NH_4)_{0.64}K_{0.36}$][Sc(aazta)(H_2O)]}·4H_2O

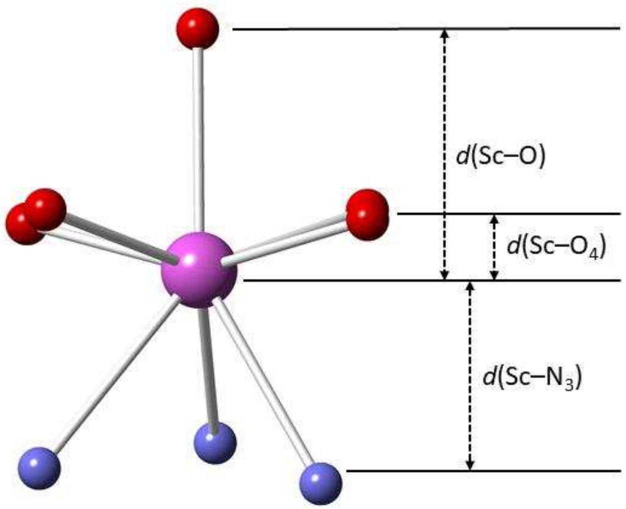

[2] G. Nagy, D. Szikra, G. Trencsenyi, A. Fekete, I. Garai, A. M. Giani, R. Negri, N. Masciocchi, A. Maiocchi, F. Uggeri, I. Toth, S. Aime, G. B. Giovenzana, Z. Baranyai; AAZTA: An Ideal Chelating Agent for the Development of ^{44}Sc PET Imaging Agents *Angew. Chem. Int. Ed.* **2017**, *56*, 2118–2122.

Table S3. Experimental crystallographic data of the Sc^{III} complexes

Parameter	$[Sc\{Sc(\mathbf{nota})\}_6]$ $Cl_3{\cdot}26H_2O$	$Li_3[\{Sc(\mathbf{L}^1)\}_6]_2$ $(ClO_4)_{10}Cl_5{\cdot}6Me_2CO$ $\cdot35.5H_2O$	$Li_2[\{Sc(\mathbf{nota})\}_2(O_2)]$ $\cdot10H_2O$	$[\{Sc(\mathbf{L}^1)\}_2(O_2)]$ $\cdot10H_2O$	$[\{Sc(\mathbf{L}^2)\}_2(O_2)]$ $\cdot12H_2O$	$Li_6[\{Sc(\mathbf{nota})\}_3$ $(C_2O_4)_3]{\cdot}14H_2O$	$[\{Sc(\mathbf{L}^1)\}_2(C_2O_4)]$ $\cdot10H_2O$
Formula	$Sc_7C_{72}H_{160}N_{18}O_{62}Cl_3$	$Li_3Sc_{12}C_{186}H_{419}N_{48}$ $O_{153.5}Cl_{15}$	$Li_2Sc_2C_{24}H_{56}N_6O_{24}$	$Sc_2C_{28}H_{67}N_8O_{22}$	$Sc_2C_{32}H_{80}N_8O_{24}$	$Li_6Sc_3C_{42}H_{82}N_9O_{44}$	$Sc_2C_{30}H_{68}N_8O_{24}$
M_r	2691.24	6876.76	916.54	958.82	1050.96	1593.68	1014.84
Habit	Prism	Prism	Prism	Prism	Prism	Prism_plate	Bar
Colour	Colourless	Colourless	Colourless	Colourless	Colourless	Colourless	Colourless
Crystal system	Trigonal	Triclinic	Triclinic	Monoclinic	Triclinic	Triclinic	Monoclinic
Space group	R–3 (148)	P–1 (2)	P–1 (2)	$C2/c$ (15)	P–1 (2)	P–1 (2)	$C2$/c (15)
a [Å]	14.3448(5)	16.6503(6)	7.9502(4)	21.9084(9)	10.1938(3)	11.4068(6)	23.8272(12)
b [Å]	14.3448(5)	20.1471(8)	11.4713(6)	16.2505(7)	10.5874(3)	18.0380(11)	16.0554(8)
c [Å]	51.592(2	26.7084(10)	11.6542(6)	15.7530(11)	11.3745(3)	18.4912(12)	15.2597(8)
α [°]	90	109.1320(10)	97.535(2)	90	87.0930(10)	61.422(2)	90
β [°]	90	98.1900(10)	107.843(2)	128.9770(10)	79.5740(10)	89.071(2)	127.361(2)
γ [°]	120	107.4270(10)	105.487(2)	90	88.8000(10)	87.658(2)	90
V [Å^3]	9194.0(8)	7783.0(5)	948.36(9)	4360.0(4)	1205.69(6)	3338.3(3)	4639.9(5)
Z	3	1	1	4	1	2	4
ρ [g·cm^{-3}]	1.458	1.467	1.605	1.461	1.447	1.585	1.453
μ [mm^{-1}]	0.530	0.478	0.457	0.400	0.371	0.409	0.383
Unique reflections	5078	35678	4720	5033	5958	15272	5336
Obsd. reflections [$I > 2\sigma(I)$]	5010	30954	4541	4514	5721	14214	5009
R	0.0422	0.0813	0.0265	0.0403	0.0313	0.0491	0.0400
R' [$I > 2\sigma(I)$]	0.0419	0.0724	0.0253	0.0380	0.0302	0.0450	0.0376
wR	0.1375	0.2098	0.0603	0.0995	0.0781	0.0923	0.0989
wR' [$I > 2\sigma(I)$]	0.1373	0.2011	0.0597	0.0978	0.0773	0.0907	0.0972
CCDC ref. no.	2484026	2484023	2484024	2484022	2484020	2484025	2484021

A

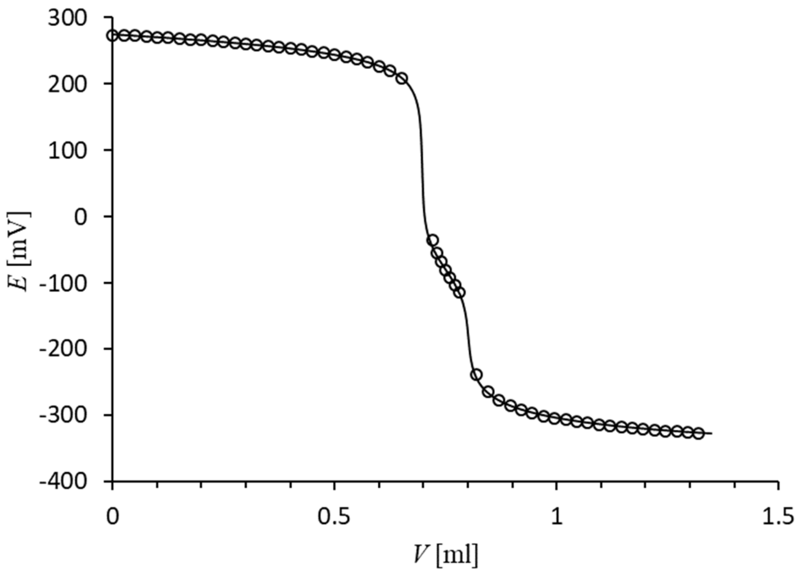

B

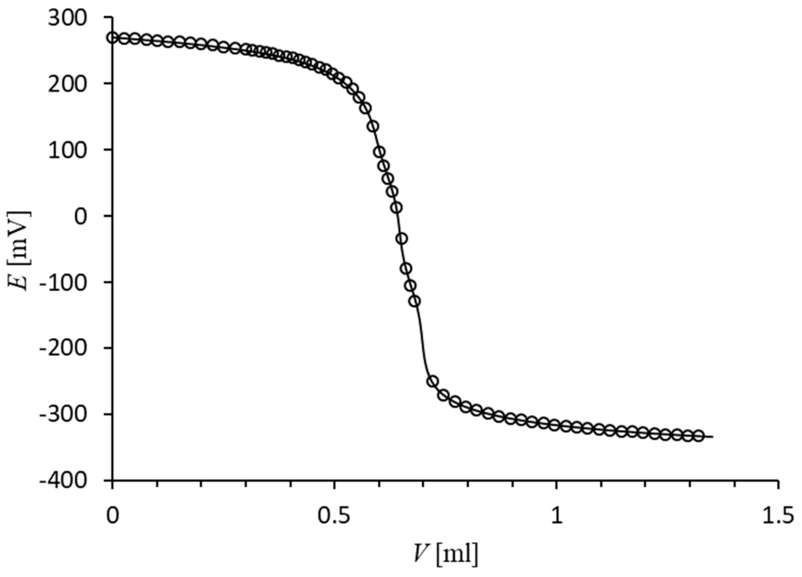

Figure S8. Examples of experimental potentiometric data (circles) and corresponding fits (lines) of Sc^{III}–H_3nota system. **A**: In-cell titration, $c_L = c_M = 4$ mM, $V = 5$ ml; **B**: In-cell titration, $c_L = 4$ mM, $c_M = 2$ mM, $V = 5$ ml. The fits were obtained by simultaneous treatment of all potentiometric data obtained for the corresponding metal-ligand system.

A

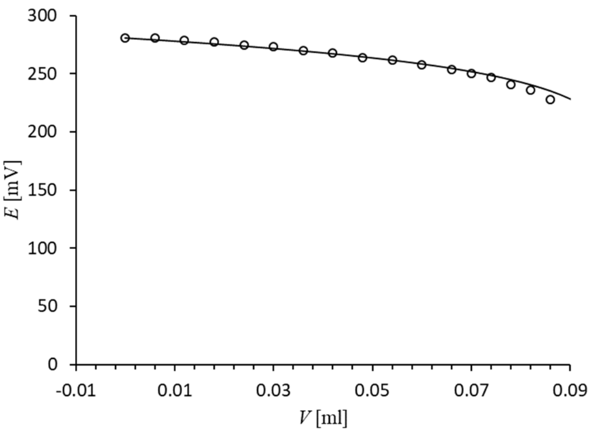

C

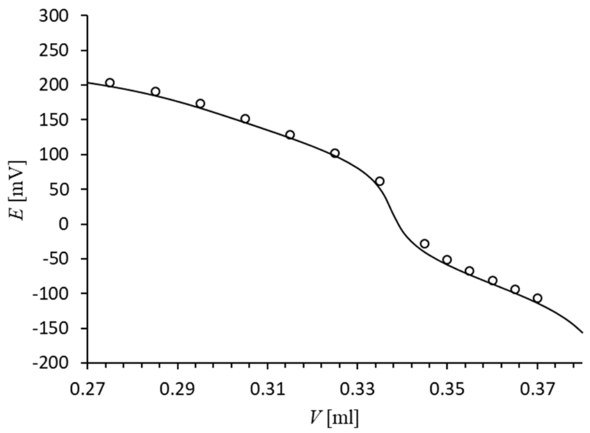

B

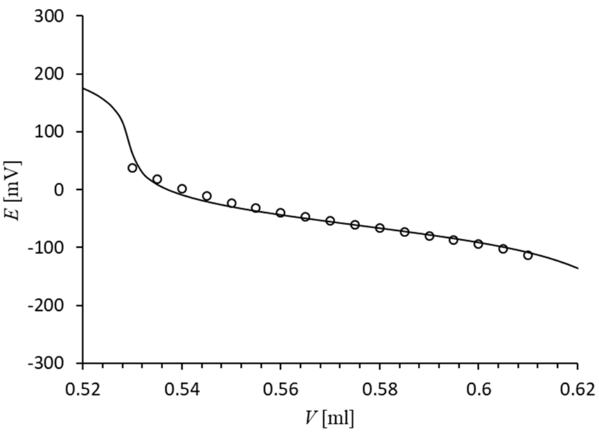

Figure S9. Examples of experimental potentiometric data (circles) and corresponding fits (lines) of Sc^{III}– H_2**L**[1] system. **A**: Out-of-cell titration of Sc^{III}–H_2**L**[1], $c_L = c_M = 4$ mM, $V = 1$ ml; **B**: In-cell titration, $c_L = c_M = 4$ mM, $V = 5$ ml; **C**: In-cell titration, $c_L = 4$ mM, $c_M = 2$ mM, $V = 5$ ml. The fits were obtained by simultaneous treatment of all potentiometric data obtained for the corresponding metal-ligand system.

Table S4. Overall stability constants (logβ) of the Sc^{III} complexes with the studied ligands (I = 0.1M $(NMe_4)Cl$, 25 °C).

	H_3nota	H_2**L**[1]	H_2**L**[2]
Equilibrium [a]	logβ	logβ	logβ
Sc + L ⇄ [Sc(L)]	19.50(1)	16.64(1)	17.94(3)
Sc + L + H_2O ⇄ [Sc(L)(OH)] + H^+	11.73(2)	9.28(1)	10.36(3)
Sc + 2 L + H^+ ⇄ [Sc(HL)(L)]	35.78(6)	32.53(2)	34.06(4)

[a] Charges are omitted.

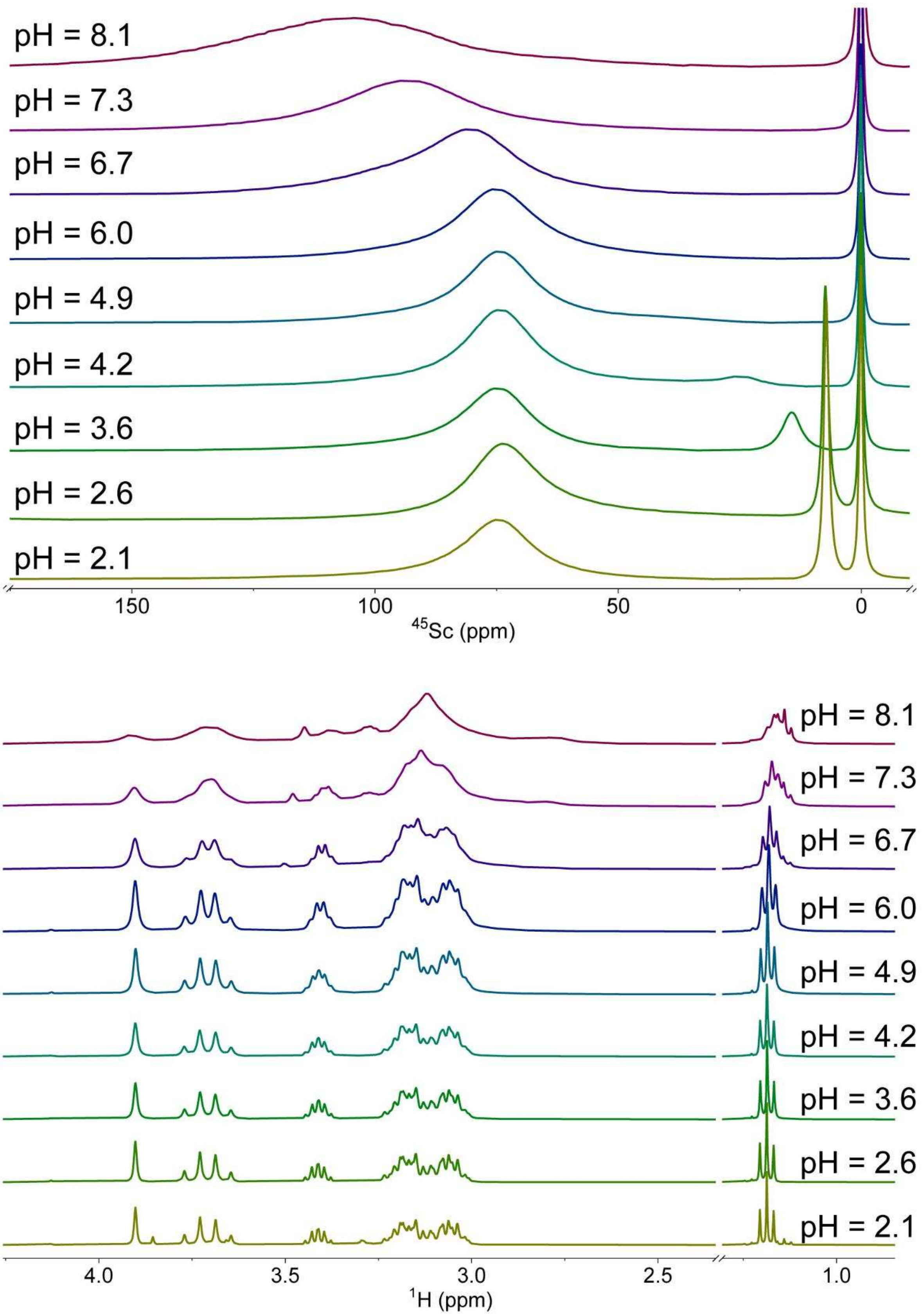

Figure S10. ^{45}Sc and ^{1}H NMR spectra of the Sc^{III}–$H_2\mathbf{L^1}$ system as a function of pH ($c_L = c_{Sc}$ = 25 mM, in D_2O, 400 MHz).

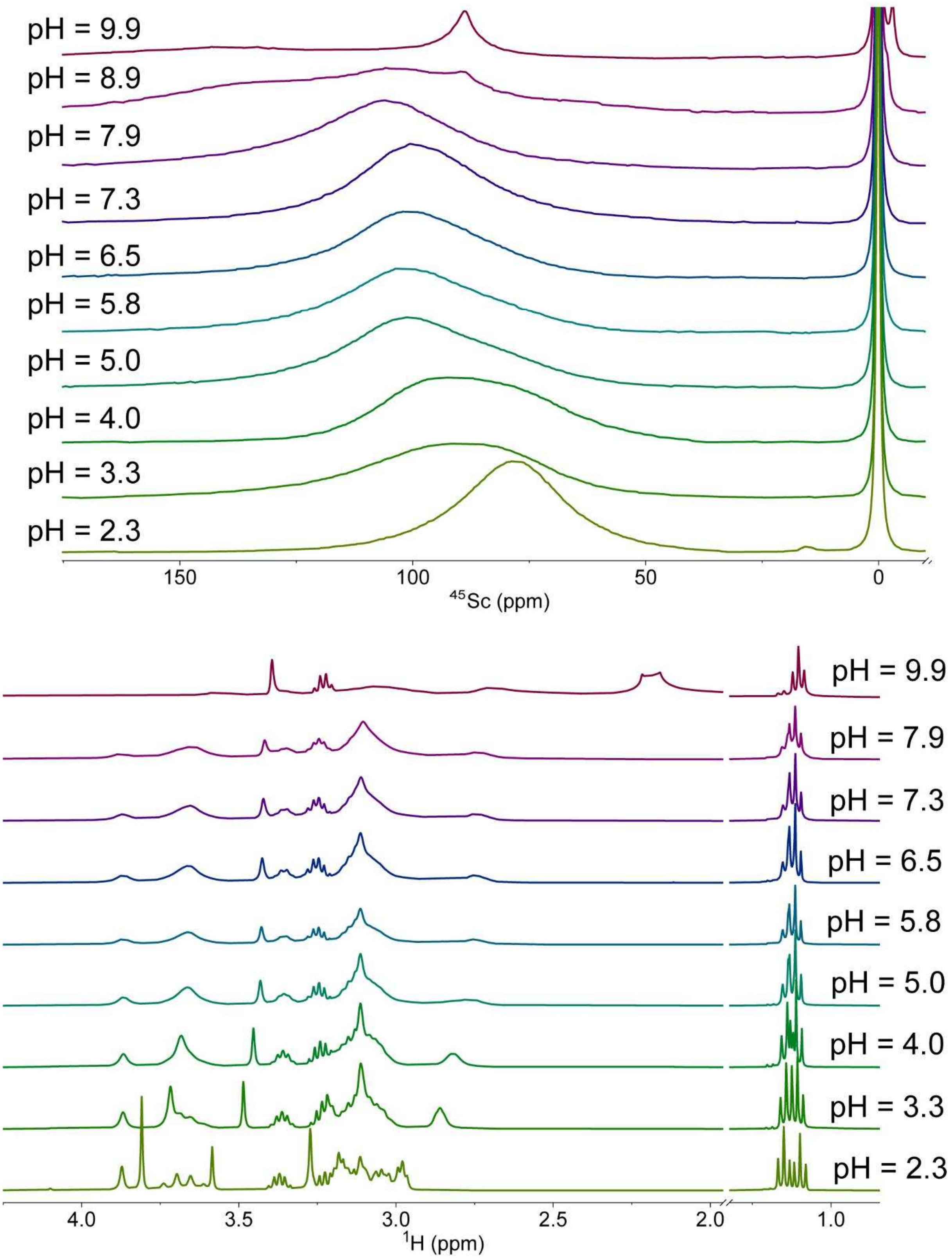

Figure S11. ^{45}Sc and ^{1}H NMR spectra of the Sc^{III}–H$_2$**L**^{1} system as a function of pH (c_L = 50 mM, c_{Sc} = 25 mM, in D$_2$O, 400 MHz).

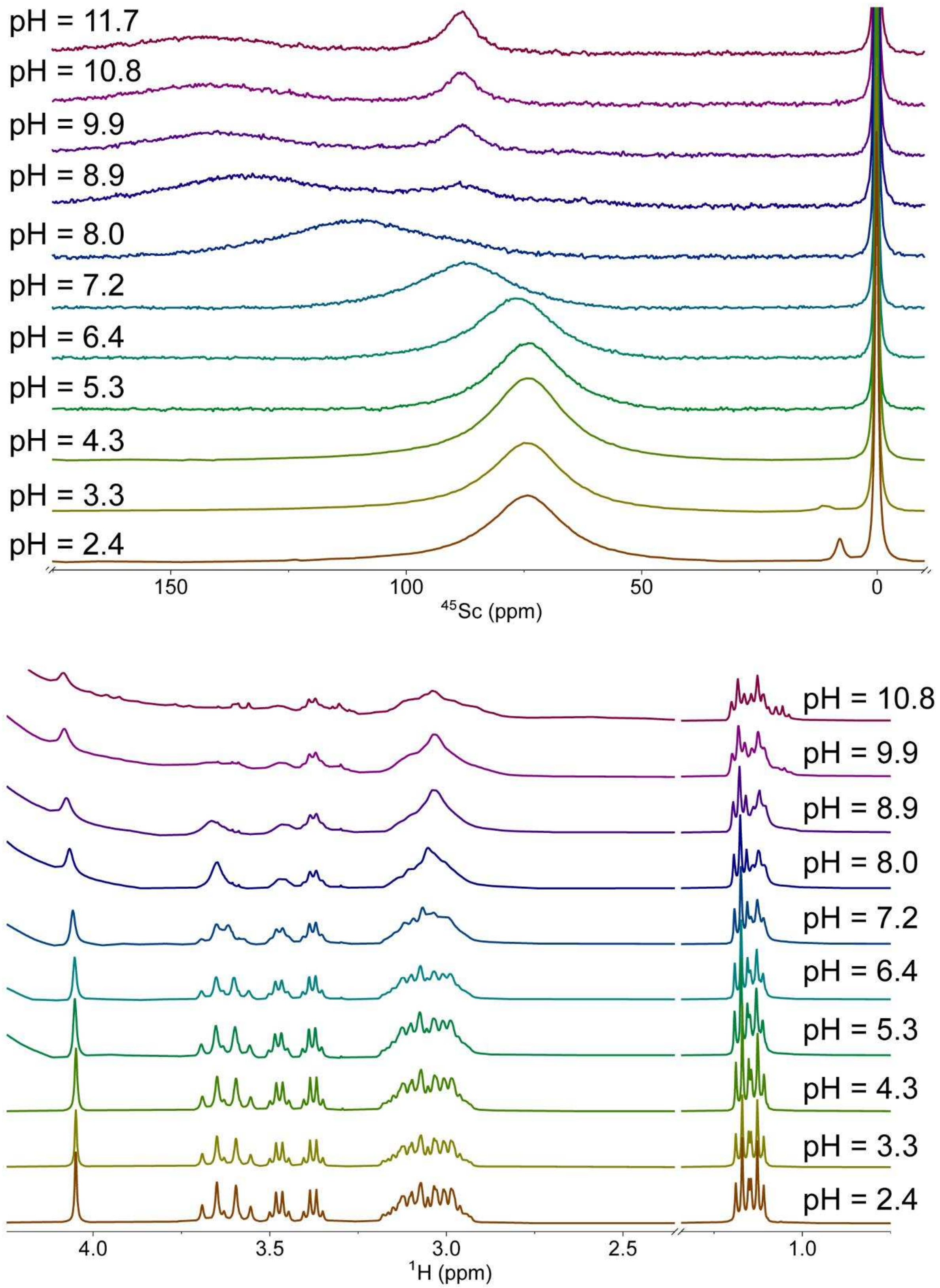

Figure S12. ^{45}Sc and ^{1}H NMR spectra of the Sc^{III}–H_2**L**$^{\mathbf{2}}$ system as a function of pH ($c_L = c_{Sc}$ = 25 mM, in D_2O, 400 MHz).

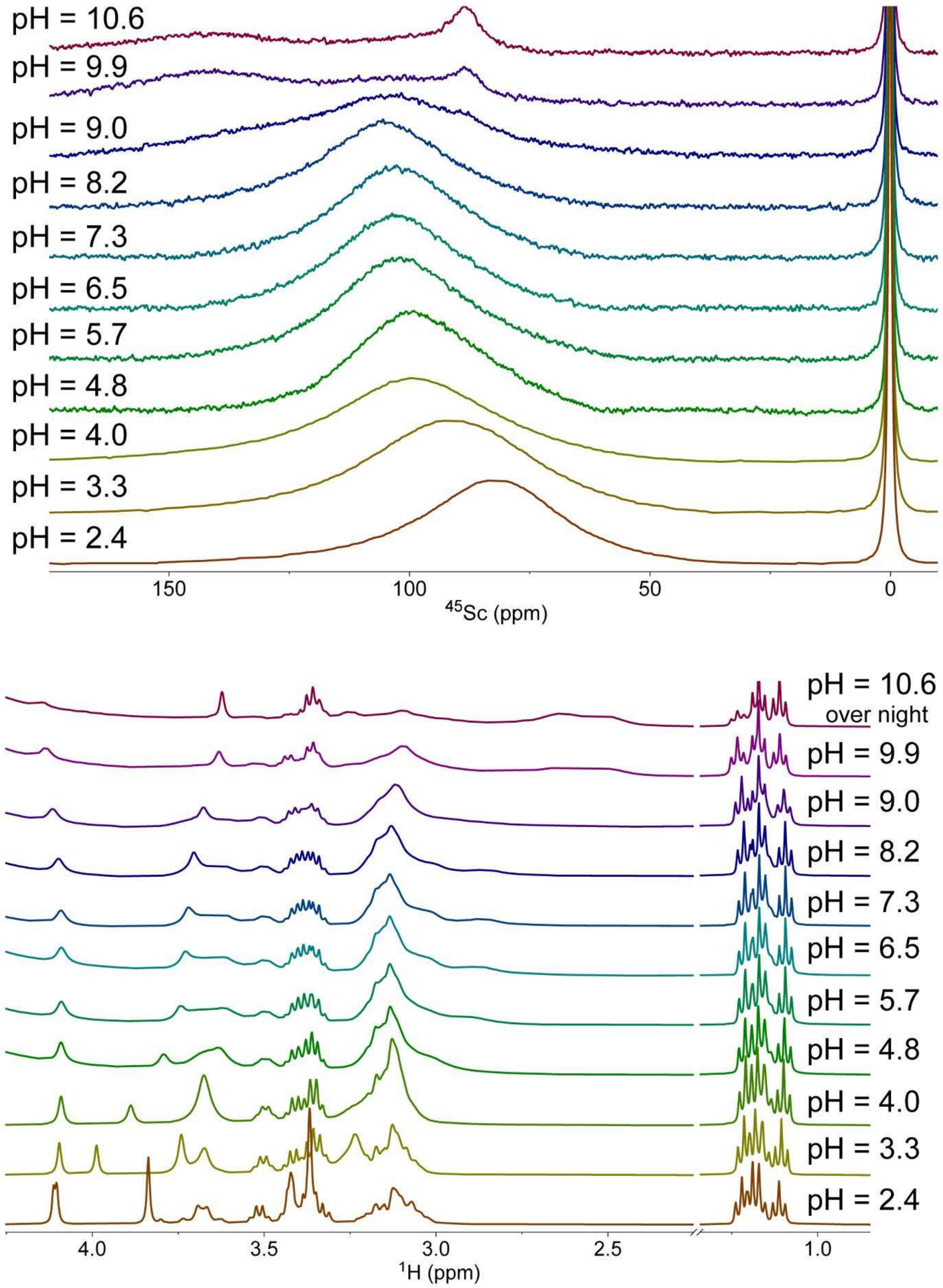

Figure S13. ^{45}Sc and ^{1}H NMR spectra of the Sc^{III}–H$_2$**L**^{2} system as a function of pH (c_L = 50 mM, c_M = 25 mM, in D$_2$O, 400 MHz).

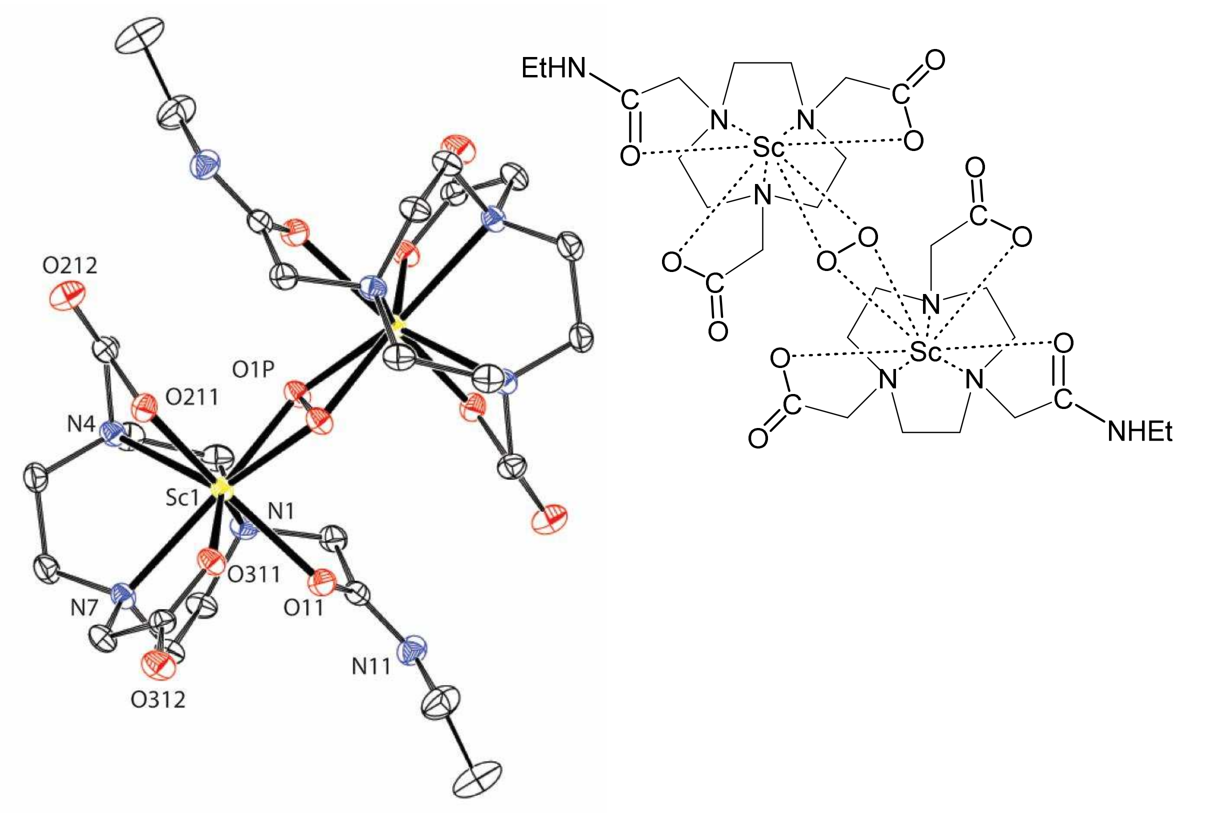

Figure S14. The molecular structure of dimeric species [(Sc($\mathbf{L^1}$)}$_2$(O_2)] found in the crystal structure of [{Sc($\mathbf{L^1}$)}$_2$(O_2)]·10H_2O. The hydrogen atoms are not shown for clarity.

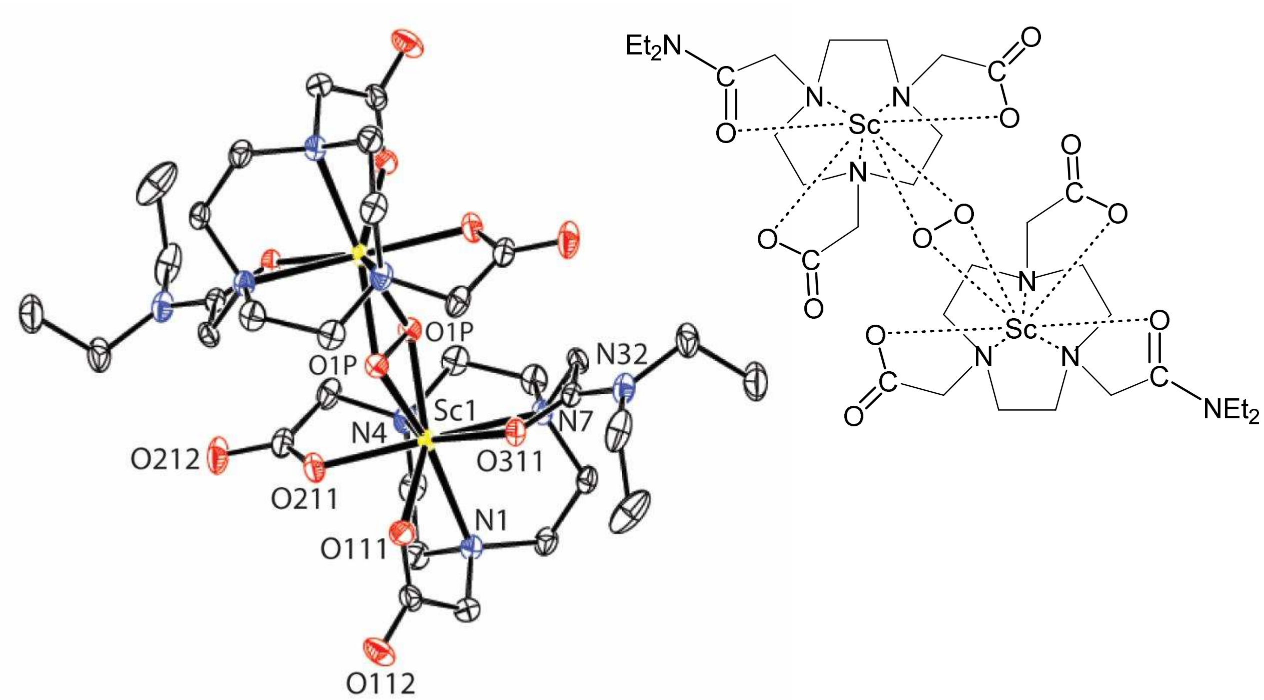

Figure S15. The molecular structure of dimeric species [{Sc($\mathbf{L^2}$)}$_2$(O_2)] found in the crystal structure of [{Sc($\mathbf{L^2}$)}$_2$(O_2)]·12H_2O. The hydrogen atoms are not shown for clarity.

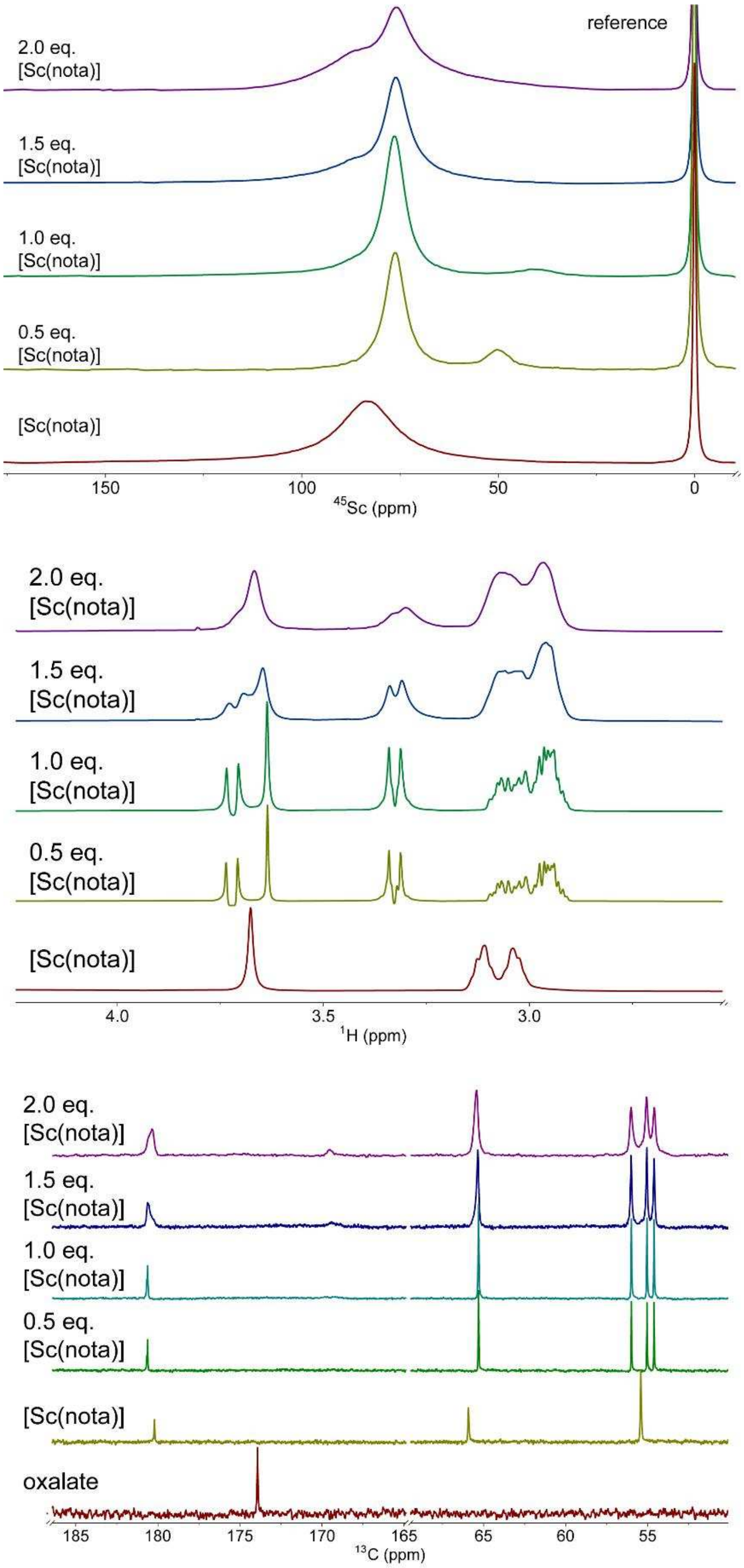

Figure S16. ^{45}Sc, ^{1}H and ^{13}C{^{1}H} NMR spectra of Sc^{III}–H$_3$nota–oxalate system as a function of the complex:oxalate ratio ($c_{oxalate}$ = 25 mM, pH 7.0, imidazole/HCl, c_{buffer} = 0.25 M, in D_2O, 400 MHz).

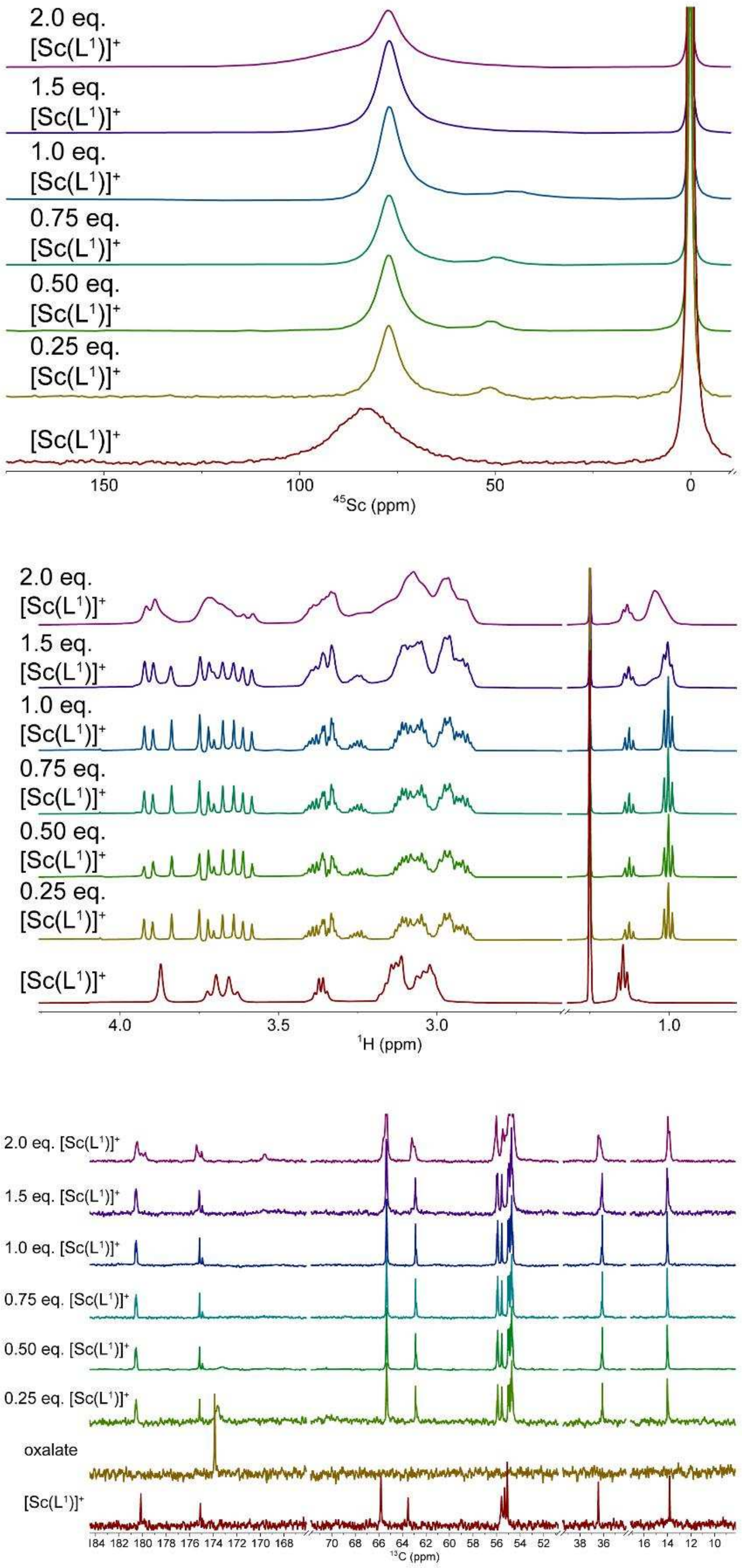

Figure S17. ^{45}Sc, ^{1}H and ^{13}C{^{1}H} NMR spectra of Sc^{III}–H$_2$**L^1**–oxalate system as a function of the complex:oxalate ratio (c_{oxalate} = 25 mM, pH 7.0, imidazole/HCl, c_{buffer} = 0.25 M, in D$_2$O, 400 MHz).

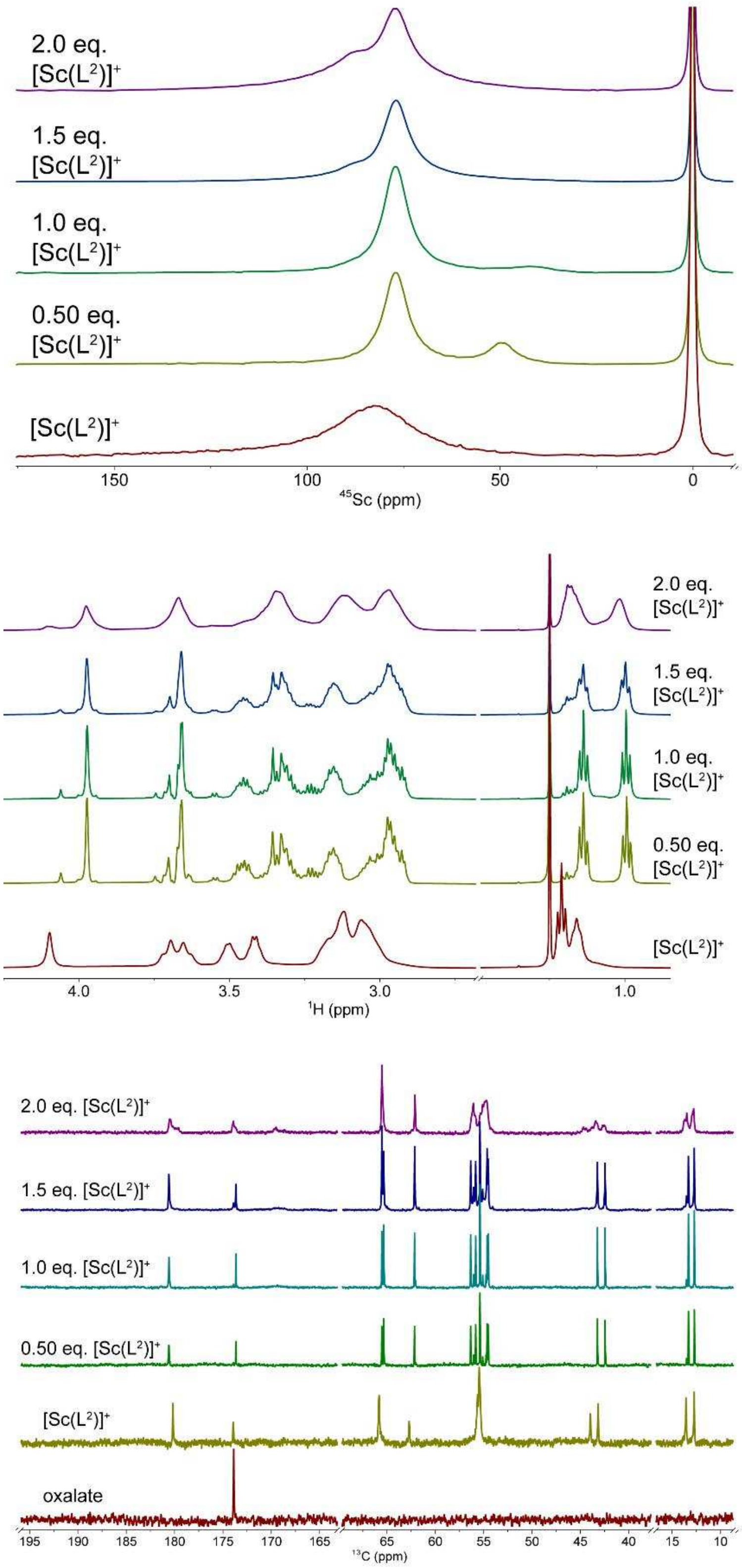

Figure S18. ^{45}Sc, ^{1}H and ^{13}C{^{1}H} NMR spectra of Sc^{III}–H_2**L**^{2}–oxalate system as a function of the complex:oxalate ratio ($c_{oxalate}$ = 25 mM, pH 7.0, imidazole/HCl, c_{buffer} = 0.25 M, in D_2O, 400 MHz).

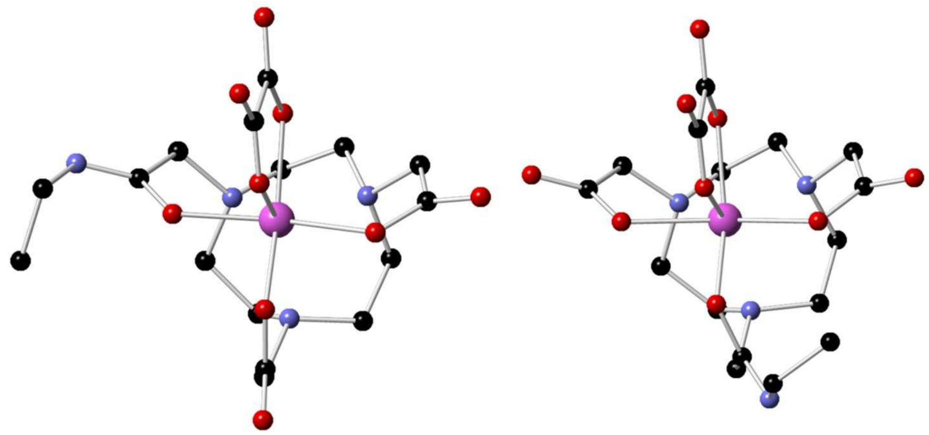

Figure S19. Calculated structures of two isomers of the ternary complex [Sc(**L**^{1})(ox)]$^{-}$ (Sc: violet, O: blue, N“ red, C: black; H atoms are not shown).
